# Restoration of Epithelial Sodium Channel Function by Synthetic Peptides in Pseudohypoaldosteronism Type 1B Mutants

**DOI:** 10.3389/fphar.2017.00085

**Published:** 2017-02-24

**Authors:** Anita Willam, Mohammed Aufy, Susan Tzotzos, Heinrich Evanzin, Sabine Chytracek, Sabrina Geppert, Bernhard Fischer, Hendrik Fischer, Helmut Pietschmann, Istvan Czikora, Rudolf Lucas, Rosa Lemmens-Gruber, Waheed Shabbir

**Affiliations:** ^1^Department of Pharmacology and Toxicology, University of Vienna Vienna, Austria; ^2^APEPTICO GmbH Vienna, Austria; ^3^Vascular Biology Center, Medical College of Georgia, Augusta University Augusta, GA, USA

**Keywords:** pseudohypoaldosteronism type 1B (PHA1B), amiloride-sensitive epithelial sodium channel (ENaC), lectin-like domain of tumor necrosis factor (TNF), TIP peptides, solnatide (AP301), AP318

## Abstract

The synthetically produced cyclic peptides solnatide (a.k.a. TIP or AP301) and its congener AP318, whose molecular structures mimic the lectin-like domain of human tumor necrosis factor (TNF), have been shown to activate the epithelial sodium channel (ENaC) in various cell- and animal-based studies. Loss-of-ENaC-function leads to a rare, life-threatening, salt-wasting syndrome, pseudohypoaldosteronism type 1B (PHA1B), which presents with failure to thrive, dehydration, low blood pressure, anorexia and vomiting; hyperkalemia, hyponatremia and metabolic acidosis suggest hypoaldosteronism, but plasma aldosterone and renin activity are high. The aim of the present study was to investigate whether the ENaC-activating effect of solnatide and AP318 could rescue loss-of-function phenotype of ENaC carrying mutations at conserved amino acid positions observed to cause PHA1B. The macroscopic Na^+^ current of all investigated mutants was decreased compared to wild type ENaC when measured in whole-cell patch clamp experiments, and a great variation in the membrane abundance of different mutant ENaCs was observed with Western blotting experiments. However, whatever mechanism leads to loss-of-function of the studied ENaC mutations, the synthetic peptides solnatide and AP318 could restore ENaC function up to or even higher than current levels of wild type ENaC. As therapy of PHA1B is only symptomatic so far, the peptides solnatide and AP318, which directly target ENaC, are promising candidates for the treatment of the channelopathy-caused disease PHA1B.

## Introduction

The amiloride-sensitive epithelial sodium channel (ENaC), a member of the ENaC/degenerin (ENaC/DEG) family of ion channels (Canessa et al., 1993; Lingueglia et al., 1993; Kellenberger and Schild, 2015), is responsible for the maintenance of Na^+^ balance, extracellular fluid volume and blood pressure (Garty and Palmer, 1997). ENaC is located at the apical membrane of salt-reabsorbing tight epithelia of the distal nephron, the distal colon, salivary and sweat glands and the lung, where it constitutes the rate-limiting step for vectorial movement of Na^+^ ions from the luminal side into the cell interior; the basolaterally located Na,K-ATPase actively transports Na^+^ out of the cell, providing the driving force for Na^+^ reabsorption (Kellenberger and Schild, 2002). In the kidney and the colon, Na^+^ reabsorption through ENaC, stimulated by the mineralocorticoid hormone aldosterone, is crucial for the maintenance of blood Na^+^ and K^+^ levels and their homeostasis (Kellenberger and Schild, 2002). In the lung, Na^+^ transport through apically-located ENaC in the alveolar epithelium is crucial for maintaining the correct composition and volume of alveolar lining fluid, enabling optimal gas exchange (Matalon et al., 2002).

The number of channels in the apical membrane depends on a balance between the rates of ENaC insertion into and removal from the membrane (Malik et al., 2006). In the distal nephron and collecting tubules of the kidney, aldosterone increases the number of active ENaC channels at the cell surface via two pathways: induction of *de novo* synthesis of ENaC subunits and reduction of ENaC retrieval from the membrane and degradation. Binding of aldosterone to the mineralocorticoid receptor results in formation of a complex which migrates to the nucleus and induces transcription of ENaC subunit genes. Aldosterone has a more immediate effect on ENaC membrane abundance through induction of the serum- and glucocorticoid-regulated kinase 1 (SGK-1), which phosphorylates the E3 ubiquitin ligase, Nedd4-2, preventing its binding to consensus proline-rich PPxY motifs in the carboxyl terminal domain of ENaC subunits, thus disrupting channel ubiquitination and subsequent internalization (Snyder, 2005; Butterworth et al., 2009). Evidence also exists for Nedd4-2-mediated regulation of ENaC membrane abundance in lung epithelia (Boase et al., 2011; Gille et al., 2014; Han and Mallampalli, 2015). In the colon, ENaC-mediated Na^+^ absorption from the intestinal lumen is under glucocorticoid control (Schild, 2010).

Four types of homologous subunit have been observed to constitute ENaC: α, β, γ, and δ (Canessa et al., 1994; Waldmann et al., 1995). A functional, pore-forming channel usually comprises one or two α- or δ-subunits, together with a β- and a γ-subunit (Canessa et al., 1994; McNicholas and Canessa, 1997). Following the determination of the trimeric structure of the ENaC homolog ASIC1 (Jasti et al., 2007), it has been widely accepted that ENaC forms a heterotrimer, although evidence for tetrameric as well as trimeric assemblies exists (Firsov et al., 1998; Kosari et al., 1998; Snyder et al., 1998; Anantharam and Palmer, 2007; Stewart et al., 2011).

Each subunit has a large extracellular loop separating two transmembrane domains with short cytoplasmic N- and C-termini (Canessa et al., 1994; Snyder et al., 1994). Alpha-ENaC is expressed in epithelial tissues of the colon, kidney and lung, whereas δ-ENaC is expressed in non-epithelial tissues of the brain, eye, pancreas, ovary and testis (Waldmann et al., 1995; Krueger et al., 2012). In lung epithelial cells, ENaC which are highly, moderately and non-selective for Na^+^ compared to K^+^ ions, respectively, have been characterized (Jain et al., 1999, 2001).

Inherited diseases associated with mutations in ENaC which increase or decrease channel activity testify to the important role of ENaC in salt and water homeostasis. Liddle's syndrome (OMIM: 177200), is a severe form of hypertension associated with ENaC hyperfunction brought about by mutations in the PPxY motif within the β- and γ- subunits (Shimkets et al., 1994; Hansson et al., 1995; Schild et al., 1995; Snyder et al., 1995; Inoue et al., 1998), and pseudohypoaldosteronism type 1B (PHA1B) (OMIM:264350), a rare, life-threatening, salt-wasting syndrome caused by decreased ENaC function (Chang et al., 1996; Strautnieks et al., 1996; Gründer et al., 1997; Boiko et al., 2015). Mouse models of PHA1B have confirmed the critical importance of individual ENaC subunits for fluid and salt regulation in the lung and distal nephron. Alpha-ENaC knockout mice die soon after birth from respiratory failure caused by severely defective alveolar fluid clearance (Hummler et al., 1996); whereas β- and γ-ENaC knockout mice die slightly later due to severe electrolyte imbalance and hyperkalemia (Hummler et al., 1997; Barker et al., 1998; McDonald et al., 1999; Pradervand et al., 1999; Bonny and Hummler, 2000). Patients suffering from PHA1B usually present in the first days of life with failure to thrive, dehydration, low blood pressure, anorexia and vomiting. Although hyperkalemia, hyponatremia and metabolic acidosis in these patients suggest hypoaldosteronism, their plasma aldosterone and renin activity are high. Children with PHA1B suffer from respiratory tract infections, wheezing and persistent rhinorrhea and some patients carrying mutations in the α-ENaC gene have markedly increased liquid volume in airway epithelia (Kerem et al., 1999; Thomas et al., 2002). PHA1B does not improve with age and patients are at risk from life-threatening, salt-losing crises, combined with severe hyperkalemia and dehydration throughout their entire lives (Zennaro and Lombès, 2004; Riepe, 2009).

The synthetically produced cyclic peptides solnatide (Lucas et al., 1994) and its congener AP318 (Hazemi et al., 2010), whose molecular structures mimic the lectin-like domain of human tumor necrosis factor (TNF), have been shown to activate ENaC in various cell- and animal-based studies (Shabbir et al., 2013, 2015; Tzotzos et al., 2013). With the aim of investigating whether the ENaC-activating effect of solnatide and AP318 could rescue loss-of-function phenotype of ENaC carrying mutations observed to cause PHA1B, Na^+^ currents elicited from mutant ENaC in the presence of the peptides were measured in whole-cell patch clamp experiments. The effect of the peptides on membrane abundance of the mutant ENaCs was analyzed in Western blotting experiments. Remarkably, both solnatide and AP318 rescued loss-of-function ENaC in all mutants tested, restoring the amiloride-sensitive Na^+^ current to physiological levels or even higher.

## Materials and methods

### Cell culture

Human embryonic kidney HEK-293 cells (ATCC no. CRL-1573) in passages 3–25, were seeded in Dulbecco's modified Eagle medium/F12 nutrient mixture Ham plus L-glutamine (DMEM/F-12; Gibco™ by Life Technologies, LifeTech Austria), supplemented with 10% fetal bovine serum (FBS; Gibco™ by Life Technologies, LifeTech Austria) and 1% penicillin-streptomycin (Sigma-Aldrich, Vienna, Austria). Cells were maintained at 37°C with 5% CO_2_ in a humidified incubator.

### Molecular biological methods

cDNAs encoding α-, β-, and γ-hENaC were a kind gift from Dr. Peter M. Snyder (University of Iowa, Carver College of Medicine, Iowa City, USA).

#### Site-directed mutagenesis

Point mutations and single base (insertions or) deletions, causing a frameshift, were introduced into cDNA encoding α-, β-, or γ-hENaC using QuikChange Lightning Site-Directed Mutagenesis Kit (Agilent Technologies, CA, USA). Pairs of mutagenic primers were designed individually with the Primer Design Program provided on the producer's website. The same bases were changed as reported in patients or at least resulting in the same protein products. The designed primers were ordered after checking their properties using the DNA Oligos Design Tool from Sigma-Aldrich, Vienna, Austria (Table 1).

**Table 1:** **Pairs of mutagenic primers for nine PHA1B mutations in conserved regions of α-, β-, and γ-hENaC**.

**Mutation**		**Primer sequence (5′-3′)**
Alpha Q101K	Forward:	CATGATGTACTGGAAATTCGGCCTGC
	Reverse:	GCAGGCCGAATTTCCAGTACATCATG
Alpha C133Y	Forward:	CGCAGTGACCATCTACACCCTCAATCCC
	Reverse:	GGGATTGAGGGTGTAGATGGTCACTGCG
Alpha S243P	Forward:	GACATACTCTCCAGGGGTGGATGCGG
	Reverse:	CCGCATCCACCCCTGGAGAGTATGTC
Alpha G327C	Forward:	GGAATCAACAACTGTCTGTCCCTGATGC
	Reverse:	GCATCAGGGACAGACAGTTGTTGATTCC
Alpha S562L	Forward:	GGTTCGGCTCCTTGGTGTTGTCTGTGG
	Reverse:	CCACAGACAACACCAAGGAGCCGAACC
Alpha S562P	Forward:	GGTTCGGCTCCCCGGTGTTGTCTG
	Reverse:	CAGACAACACCGGGGAGCCGAACC
Alpha S243fs	Forward:	GACATACTCATCGGGGTGGATGCGG
	Reverse:	CCGCATCCACCCCGATGAGTATGTC
Beta G37S	Forward:	CACCAACACCCACAGCCCCAAGCGCAT
	Reverse:	ATGCGCTTGGGGCTGTGGGTGTTGGTG
Gamma V543fs	Forward:	GGATGAGCTGTTCTTTGTCTGCGTCATCG
	Reverse:	CGATGACGCAGACAAAGAACAGCTCATCC

Mutant strands were synthesized by PCR with a *Pfu*-based DNA polymerase using 100 ng wild type (WT) cDNA encoding α-, β-, or γ-hENaC and the specific mutagenic primer pair. Parental methylated and hemi-methylated template DNA was digested with a *Dpn I* restriction enzyme for 5 min at 37°C. Transformation was performed with XL10-Gold ultracompetent cells provided with the kit. Aliquots of the cells were treated with β-mercaptoethanol for 2 min and incubated with the amplified DNA for 30 min on ice. After 30 s of heat pulse at 42°C and 2 min on ice, cells were grown in NZY^+^ broth (10 g NZ amine (casein hydrolysate), 5 g yeast extract, 5 g NaCl, ddH_2_O added to a final volume of 1 l, adjusted to pH 7.5 using NaOH, autoclaved, addition of filter-sterilized 12.5 ml 1 M MgCl_2_, 12.5 ml 1 M MgSO_4_, 10 ml 2 M glucose) for 1 h at 37°C with shaking at 250 rpm. Cells were centrifuged at 800 × g for 2 min and the majority of the supernatant was discarded. The cell pellet was resuspended in the remaining media and all cells were spread on 60 mm LB agar (20 g LB broth, 15 g agar, ddH_2_O added to a final volume of 1 l, autoclaved) plates containing 100 μg/ml ampicillin (the appropriate antibiotic for the plasmid vector) and incubated overnight (16–18 h) at 37°C. Single colonies were picked and cultured in LB broth (20 g LB broth, ddH_2_O added to a final volume of 1 l, autoclaved) supplemented with 100 μg/ml ampicillin overnight (16–18 h) at 37°C with shaking at 250 rpm. 2 ml of the bacterial culture were used for DNA extraction; the remaining cell suspension was mixed well with the same volume of glycerol (Sigma-Aldrich, Vienna, Austria) and stored at −80°C in order to obtain a glycerol stock.

The DNA was extracted from *Escherichia coli* (*E. coli*) cells with the GeneJET Plasmid Miniprep Kit (Thermo Scientific Loughborough, UK) according to the producer's protocol. The cell pellet from 2 ml overnight culture was resuspended, lysed and neutralized. The supernatant was loaded to the column, washed twice and the plasmid DNA was eluted. The DNA concentration was measured with NanoDrop ND-1000 Spectrophotometer by threefold determinations. In order to verify the presence of the mutation the extracted DNA was sent to LGC Genomics GmbH, Berlin, Germany, for sequencing. The sequencing result was evaluated with the online available ExPASy Translate tool (http://web.expasy.org/translate/).

#### Midi prep

Glycerol stocks of *E. coli* colonies carrying positively mutated cDNA were picked and cultivated in LB broth supplemented with 100 μg/ml ampicillin. For larger amounts of DNA the Plasmid Midi Kit (QIAGEN GmbH, Hilden, Germany) was used. The cell pellet from 100 ml overnight culture was resuspended, lysed and neutralized. The supernatant was loaded to the equilibrated column, washed twice and the plasmid DNA was eluted. The DNA was precipitated with isopropanol and the DNA pellet was washed with 70% ethanol. The dry DNA pellet was dissolved in elution buffer; the DNA concentration was measured with NanoDrop ND-1000 Spectrophotometer and diluted to a concentration of about 600 ng/μl.

#### Transfection

HEK-293 cells were transfected 1 day after cell seeding using X-tremeGENE HP DNA transfection reagent (Roche Diagnostics, Mannheim, Germany) according to the manufacturer's protocol. A set of one mutant subunit and the other two WT subunits of hENaC (250 ng cDNA per subunit for a 35 mm cell culture dish) was used for each experiment and the ratio of DNA to transfection reagent was 1:3. The expression was highest 48 to 72 h after transfection.

### Cell surface biotinylation

HEK-293 cells were cultured in 100 mm dishes in 5% CO_2_ incubator at 37°C in DMEM medium supplemented with 5% FBS. Cells were harvested after reaching 90% confluency. Solnatide and AP318 were added at a final concentration of 200 nM into the medium, and the cells were incubated for 5 or 10 min. Then cells were washed 2× with 10 ml cold phosphate buffered saline (PBS), then treated with 10 ml cold PBS containing 2.5 mg EZ-Link Sulfo-NHS-SS-Biotin (Thermo Scientific, Rockford, USA), and incubated on ice with light agitation for 30 min. Fifty ml quenching solution were added to the cells. Cells were scraped and transferred to fresh 50 ml tube. The flask was rinsed with 10 ml Tris-buffered saline (TBS), and also the rinsed volume was transferred to the present 50 ml tube. Cell suspension was centrifuged at 500 × g for 3 min and supernatant was discarded. Five ml TBS was added to the cell pellet. The cell pellet was resuspended, cell suspension was centrifuged at 500 × g for 3 min, and also the supernatant was discarded. Antipeptidase cocktail (10 μM pepstatin A, 10 μM phenylmethylsulfonyl fluoride and 10 μM leupeptin) was added to lysis buffer that was applied to the cell pellet. Cells were resuspended, transferred to 1.5 ml microcentrifuge tube so homogenized on ice by ultrasonication using 1 s bursts. Cell material was incubated on ice for 30 min. Intact cells and nuclei were pelleted from the cell material by 10,000 × g for 2 min at 4°C, processed supernatant was transferred to a fresh tube and incubated overnight at 4°C with 0.5 ml NeutrAvidin Agarose. Then washed 3× with cell lysis buffer. The biotinylated proteins were eluted with 100 μl sodium dodecyl sulfate (SDS) sample buffer (62.5 mM Tris, pH 6.8, 1% SDS, 10% glycerine, 50 mM dithiothreitol) containing 10 mM leupeptin. Trace amounts of bromophenol blue were added to the eluates, and these eluates were analyzed after heating for 10 min at 65°C by SDS-PAGE and immunoblotting.

### Western blotting

Corresponding amounts of protein were separated under reducing conditions by SDS-PAGE on 7.5% SDS gels along with color-coded prestained protein marker (cat. #12949 from Cell Signaling). Proteins were transferred onto a nitrocellulose membrane (UltraCruz™ 0.45 mm, Cat. # sc-3723 Santa Cruz Biotechnology, Texas U.S.A) by tank blotting (Mini Trans-Blot® Transfer System, Bio-Rad Laboratories, Vienna, Austria) at 100 V for 60 min. Non-specific binding sites were blocked by incubation with 3% bovine serum albumin in PBS, supplemented with 0.02% sodium azide, 1 h in room temperature and/or overnight at 4°C. Then the membranes were incubated with primary antibody solutions (anti-α-ENaC NH2-terminal Cat. # sc-22239, anti-β-COOH-terminal sc-22242, anti-γ-internal region sc-22245 ENaC, and anti-β-tubulin antibodies from Santa Cruz Biotechnology). Subsequently, membranes were washed 3x with TBS containing 0.1% Tween 20 (TBST), and corresponding horseradish peroxidase-conjugated secondary antibodies (Santa Cruz Biotechnology) were applied. After incubation for 1.5 h at room temperature and washing four times with TBST solution, enhanced chemiluminescence (ECL) substrate (Amersham ECL Plus Western Blotting Detection Reagent, GE Healthcare, Vienna, Austria) was used for visualization. Following incubation for 2 min, membranes were exposed to X-ray films (Amersham Hyperfilm ECL, GE Healthcare). Exposed films were scanned and quantified using ImageJ (NIH, Maryland, USA).

### Electrophysiology

Whole-cell currents were recorded from transfected HEK-293 cells with an Axopatch 200B amplifier, DigiData 1440A and pCLAMP10.2 software (Axon Instruments, Union City, CA) at room temperature (19–22°C) 48–72 h after transfection. Currents were recorded at 10 kHz and filtered at 5 kHz. The chamber contained 1 ml bath solution of the following composition (in mM): 145 NaCl, 2.7 KCl, 1.8 CaCl_2_, 2 MgCl_2_, 5.5 glucose, and 10 HEPES, adjusted to pH 7.4 with 1 M NaOH solution. The borosilicate glass patch pipettes (Harvard Apparatus, Holliston, MA) had resistances of 2–4 MΩ. The pipette solution contained (in mM): 135 potassium methane sulphonate, 10 KCl, 6 NaCl, 1Mg_2_ATP, 2 Na_3_ATP, 10 HEPES, and 0.5 EGTA, adjusted to pH 7.2 with 1 M KOH solution. Data acquisition and storage were processed directly to a PC. After GΩ-seal formation, the equilibration period of 5 min was followed by recordings at a holding potential of −100 mV. Stock solutions (1 or 10 μM) of solnatide and AP318 were prepared with distilled water, and aliquots (2–20 μl) were cumulatively added into the bathing solution, to reach concentrations of 2–200 nM. Latest after 5 min steady state level was reached and the mean of 10 sweeps was taken for further data analysis before adding the next higher concentration. The current was measured continuously and the same clamp protocol was applied during control recordings, and in presence of solnatide or AP318. At the end of the experiments, 10 μM amiloride was applied to show whether the peptide-induced increase in current was due to the amiloride-sensitive Na^+^ current. The amiloride-sensitive current was calculated by subtracting the current in presence of amiloride from the current in absence of amiloride and the solnatide- and AP318-induced current was calculated by subtracting the current during control conditions from the increased current after treatment with the respective peptide. To analyze the role of glycosylation in solnatide-induced activation of ENaC carrying PHA1B mutations the membrane of transfected HEK-293 cells were deglycosylated with peptide-*N*^4^-(*N*-acetyl-β-D-glucosaminyl)asparagine amidase F (PNGase F, peptide *N*-glycanase). Cells were incubated with PNGase F (100 U) immediately prior to patch-clamp experiments, as previously described by Shabbir et al. (2013). Data was analyzed with OriginPro 2017 (OriginLab, Northampton, MA, USA) and figures were edited with CorelDRAW X7 (Corel Corporation, Ottawa, ON, Canada). Data are represented as mean ± SEM. Differences were evaluated by one-way ANOVA followed by Tukey's *post-hoc* test.

### Test compounds

**Figure 1:**
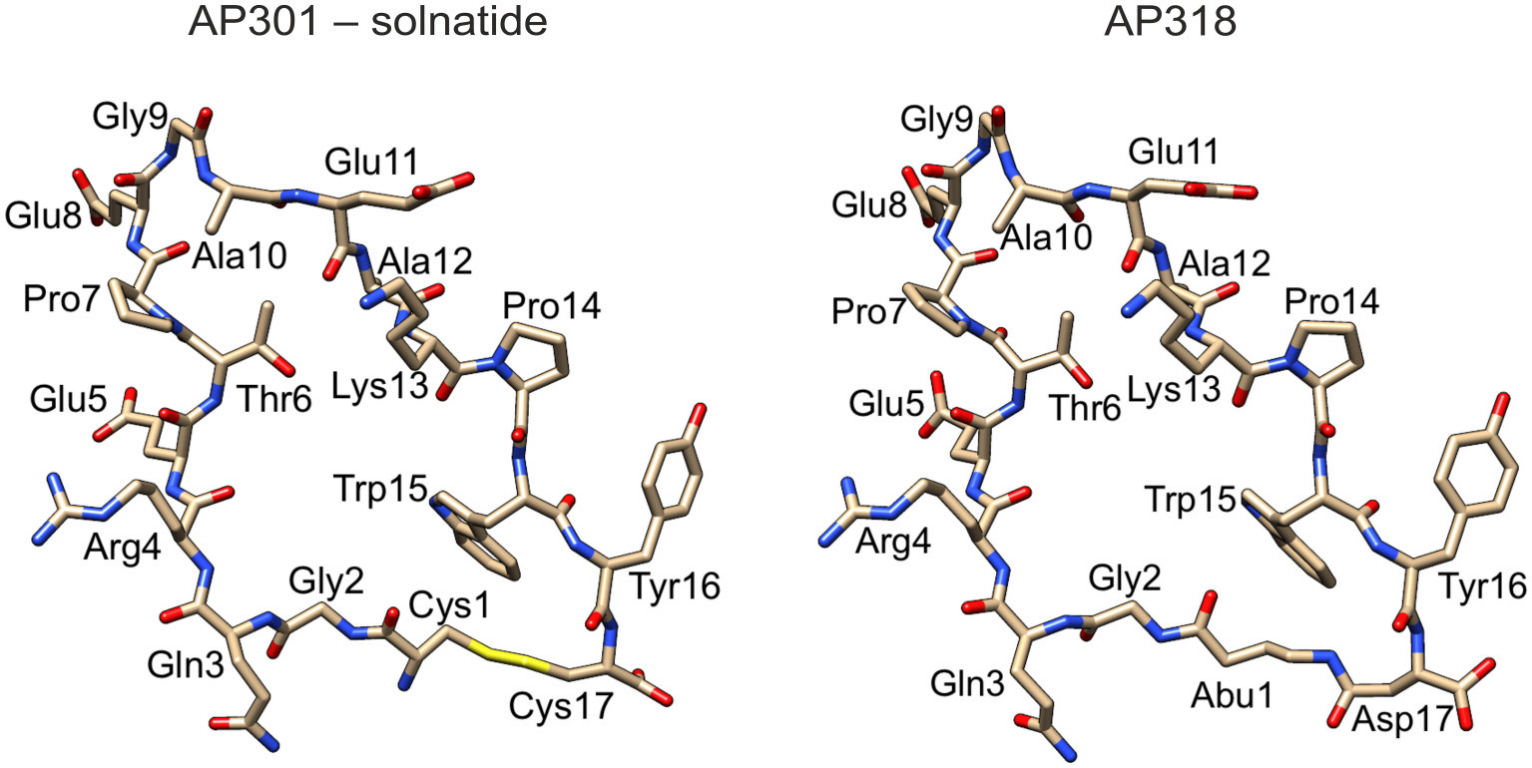
**Structure of solnatide (AP301) and AP318**. Peptides were modeled according to a previously described protocol (Hazemi et al., 2010) using the crystal structure of TNFα, PDB ID: 3L9J (Byla et al., 2010), as a template and rendered using the molecular graphics program Chimera, version 1.10.2 (Pettersen et al., 2004). Abu, 4-aminobutanoic acid.

Solnatide (CAS Registry Number: 259206-53-6; CA Index Name: L-cysteine, L-cysteinylglycyl-L-glutaminyl-L-arginyl-L-.alpha.-glutamyl-L-threonyl-L-prolyl-L-.al-pha.-glutamylglycyl-L-alanyl-L-.alpha.-glutamyl-L-alanyl-L-lysyl-L-prolyl-L-tryptophyl-L-tyrosyl-, cyclic (1.fwdarw.17)-disulphide) with the amino acid sequence CGQRETPEGAEAKPWYC, and AP318 (*Cyclo*(4-aminobutanoic acid-GQRETPEGAEAKPWYD)) are derived from the lectin-like domain of TNF (CQRETPEGAEAKPWYE), and are also called TIP peptides. Synthesis and description of the peptides is reported in detail by Hazemi et al. (2010). All chemicals, reagents and culture media were obtained from Sigma-Aldrich (Vienna, Austria), unless stated otherwise. See Figure 1 for molecular models of solnatide and AP318 (see also Hazemi et al., 2010).

## Results

The ability of the cyclic peptides solnatide and AP318 to activate ENaC harboring PHA1B-causing mutations was tested, in order to identify novel therapeutic candidates for PHA1B treatment. The only difference between solnatide and AP318 is that the disulphide bridge between cysteines in positions 1 and 17 and which links the N- and C-termini in solnatide has been replaced by an amide bond linking the amino group of 4-amino-butanoic acid in position 1 with the side chain carboxyl group of aspartic acid in position 17 (Figure 1).

Mutations in α-, β-, and γ-subunits of ENaC associated with PHA1B are described in the literature; there are no reports of PHA1B-causing mutations in δ-ENaC. In this work mutations which occur at residues conserved in all four subunits of hENaC, and which have been confirmed by genetic analysis of patients diagnosed with PHA1B, were reconstructed and studied regarding their macroscopic current and membrane abundance. Conserved regions seem to play an integral role in the functionality of the channels, which makes them appear particularly relevant to be observed in the first instance.

**Figure 2:**
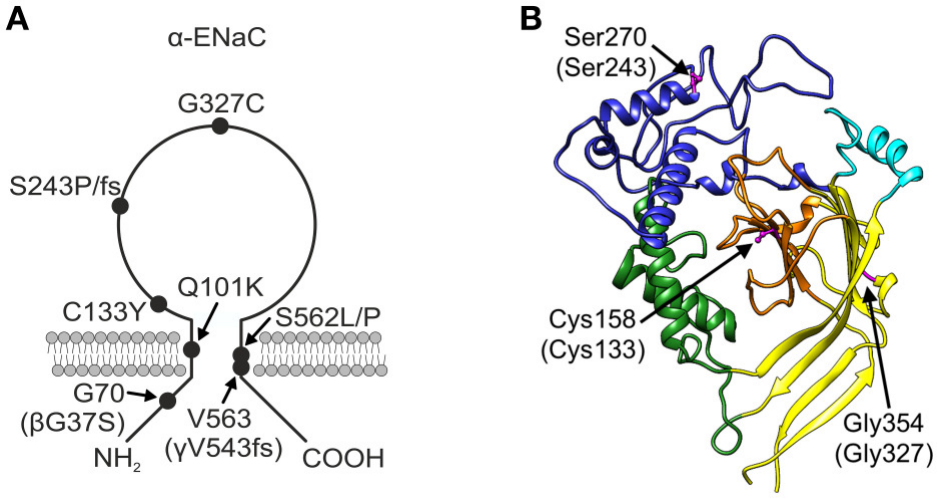
**PHA1B mutants mapped onto ENaC. (A)** Topology model of α-ENaC subunit showing approximate location of PHA1B mutations in primary sequence, and mutations in β- and γ-subunits in brackets. **(B)** Homology model of mouse α-ENaC extracellular domain (Kashlan et al., 2011), with residues equivalent to those in human α-ENaC which are the sites of PHA1B mutations highlighted (pink with ball and stick rendering of side chains); residue numbering is for mouse α-ENaC (UniProt Q61180, SCNNA_MOUSE) with equivalent residue numbers of human α-ENaC (UniProt P37088, SCNNA_HUMAN) in brackets; color of the backbone ribbon indicates the approximate location of domains in the ASIC1 structure according to Jasti et al. (2007), viz: “palm,” yellow; “β-ball,” orange; “finger,” blue; “thumb,” green; “knuckle,” cyan. Graphic created using Chimera, version 1.10.2 (Pettersen et al., 2004).

The βG37S mutation, which is equivalent to G70 in the α-subunit and has been electrophysiologically characterized before (Chang et al., 1996; Gründer et al., 1997; Kucher et al., 2011), is located intracellularly in the N-terminal region of the polypeptide chain (Figure 2A). The αQ101K mutation is located in the first transmembrane region TM1 of α-ENaC toward the extracellular side (Mora-Lopez et al., 2012). Mutations in the extracellular loop can be assigned to domains as defined by Jasti et al. (2007) for the X-ray structure of the chicken acid-sensing channel (ASIC1), a homolog of ENaC. The αC133Y mutation (Bonny et al., 1999) lies in the core of the extracellular loop, within the β-ball (colored orange in Figure 2B). Mutations αS243P (Dirlewanger et al., 2011) and αS243fs (Schaedel et al., 1999) lie in the finger (colored blue in Figure 2B), rather exposed in the extracellular loop. The frameshift at this position results in a premature stop codon only four amino acids after the affected S residue. The αG327C mutation (Edelheit et al., 2005) lies within the palm domain (colored yellow in Figure 2B) at the boundary with the β-ball and αS562L (Schaedel et al., 1999) and αS562P (Riepe et al., 2009) lie within the TM2 region. The γV543fs mutation (Adachi et al., 2001), which is equivalent to V563 in the α-subunit, is similarly located within the TM2 domain. The γV543fs mutation is predicted to result in synthesis of a truncated translation product of 597 amino acid residues with an abnormal (non-WT) sequence after S542.

**Figure 3:**
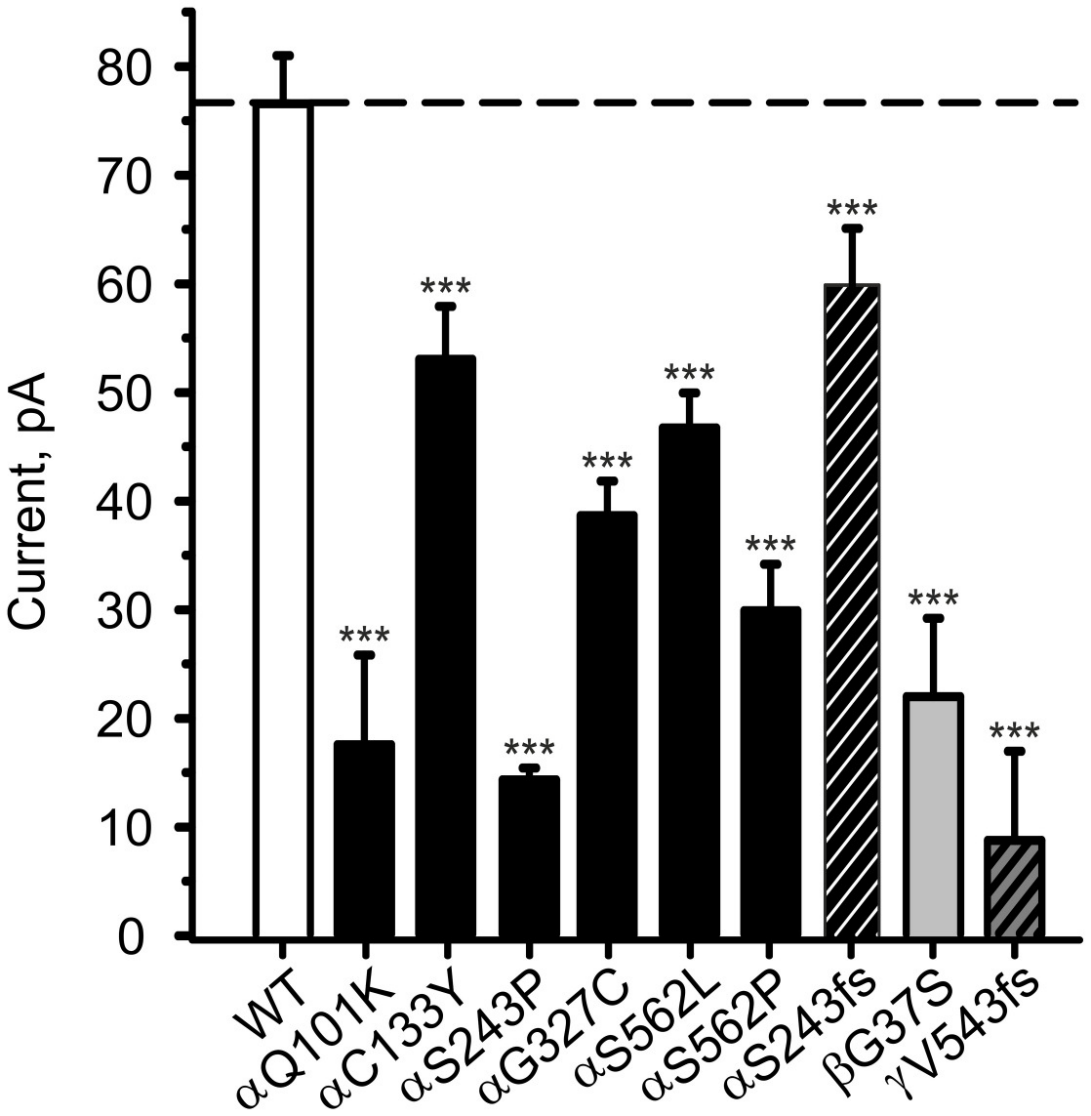
**PHA1B-causing mutations in hENaC exhibit low current**. Mean values of (10 μM) amiloride-sensitive Na^+^ current in HEK-293 cells expressing WT hENaC (empty bar) and PHA1B-causing mutations in either α- (filled or hatched, black bars), β- (filled, light gray bar) or γ- (hatched, dark gray bar) hENaC subunits are shown. Point mutations are indicated with filled bars, whereas frameshift mutations are indicated with hatched bars. WT or mutant hENaC were patched in the whole-cell mode; inward current was elicited at −100 mV. All mutants showed a significant decrease in current vs. WT ENaC, ^***^p < 0.001 (*n* = 6–13).

To study the channel function, combinations of αβγ-ENaC comprising one mutant and two WT chains were expressed in HEK-293 cells and the amiloride-sensitive Na^+^ current in the whole-cell mode was recorded. All investigated ENaC carrying PHA1B mutations showed a lower control current than WT ENaC (Figure 3), which is likely to be the cause of the disease.

All tested control and solnatide- or AP318-induced currents of HEK-293 cells transfected with WT or mutant ENaC were amiloride-sensitive and were significantly higher than whole-cell current of mock-transfected HEK-293 cells, and therefore can be identified as ENaC-current. The control current of mock-transfected cells was 11.28 ± 5.68 pA and did not change after 10 μM amiloride (10.47 ± 6.23 pA), 200 nM solnatide (11.24 ± 3.01 pA) or 200 nM AP318 (11.59 ± 1.24 pA) treatment, so the amiloride-sensitive and solnatide- or AP318-induced current of mock-transfected HEK-293 cells was about zero.

**Figure 4:**
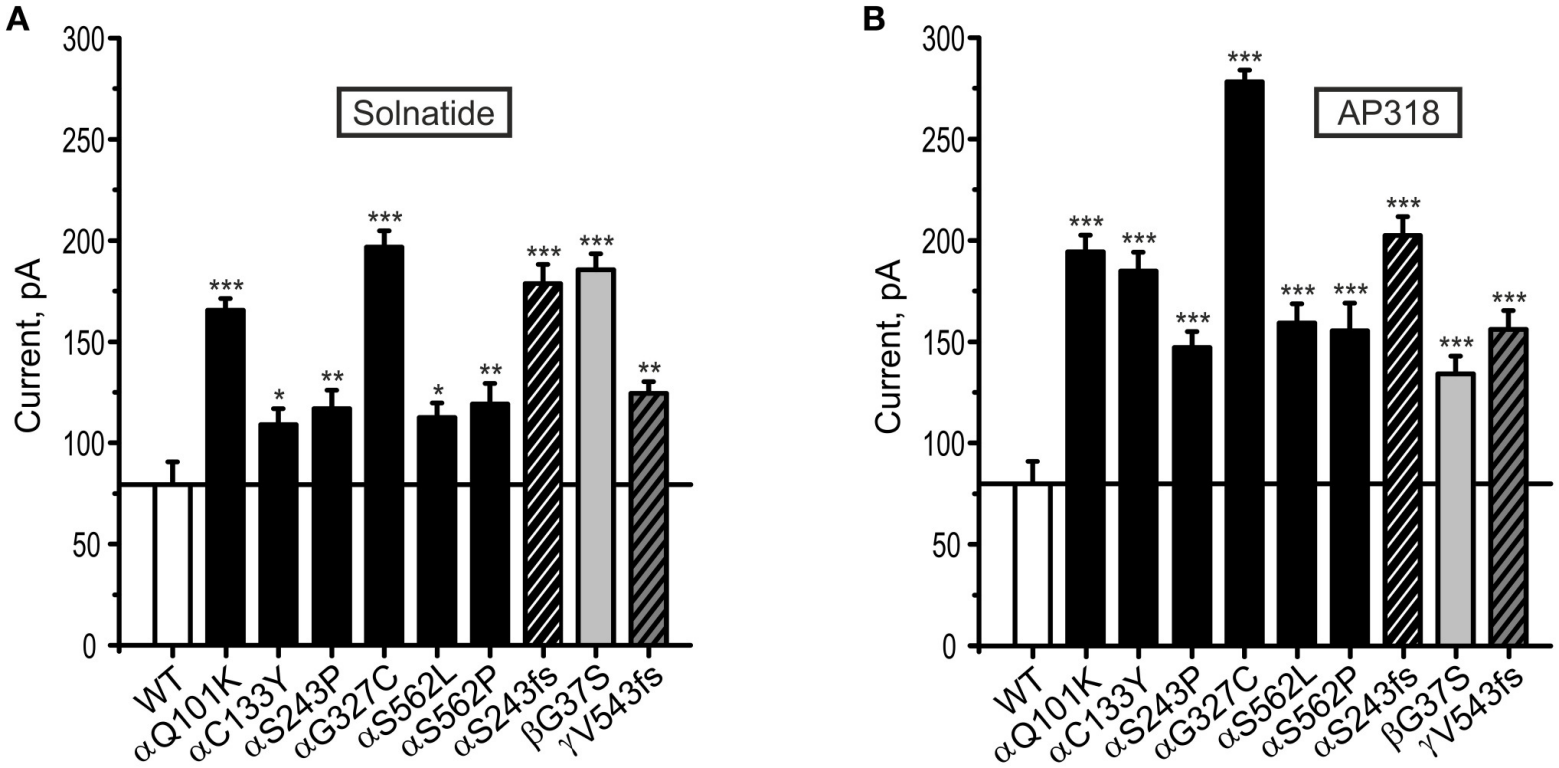
**Both solnatide and AP318 activate macroscopic current reaching to WT (control) in PHA1B mutations**. Mean data of 200 nM solnatide- **(A)** and AP318- **(B)** induced Na^+^ current in HEK-293 cells expressing hENaC harboring PHA1B-causing mutations in either α- (filled or hatched, black bars), β- (filled, light gray bar) or γ- (hatched, dark gray bar) subunits are shown. Point mutations are indicated with filled bars, whereas frameshift mutations are indicated with hatched bars. For comparison with the (10 μM) amiloride-sensitive current of WT hENaC (without treatment) (empty bar) solid line is shown. All PHA1B mutants showed currents equal to/more than WT hENaC followed by solnatide and AP318 treatments (amiloride-sensitive current of WT: *n* = 13; solnatide- and AP318-induced current of mutant ENaC: *n* = 3–8). Significant differences compared to WT control are indicated, ^*^p < 0.05, ^**^p < 0.01, ^***^p < 0.001.

**Figure 5:**
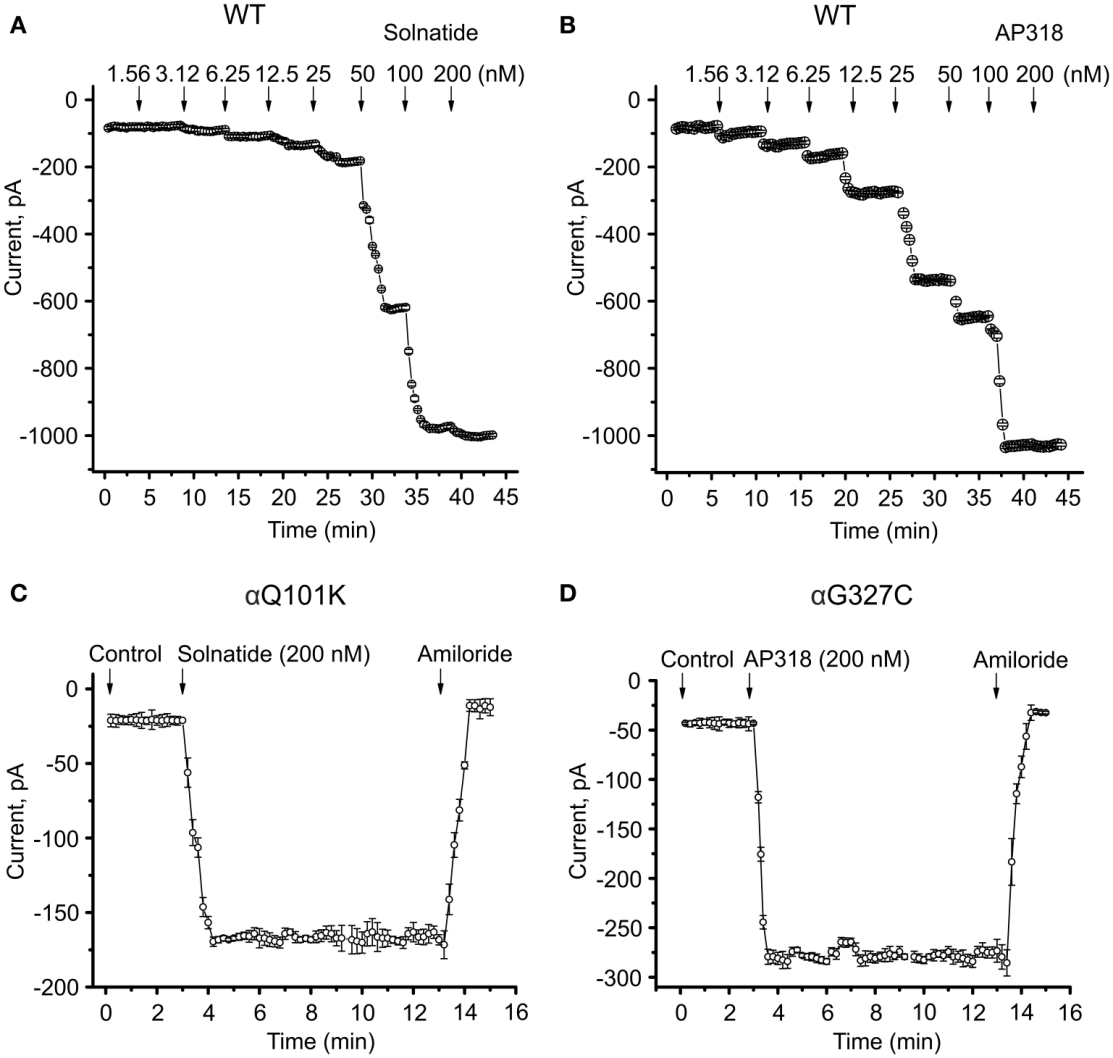
**Time course of current activation of ENaC with solnatide and AP318**. Dose-response relationship for solnatide **(A)** and AP318 **(B)** on WT ENaC is shown in a time-dependent manner. Both peptides were applied at increasing concentrations from 1.56 to 200 nM at indicated time points (arrows). For each concentration only the current amplitude during steady state levels were used for further analysis. Additionally the time course of activation of αQ101K by solnatide **(C)** and of αG327C by AP318 **(D)** is shown. The activation is rapid and consistent at least for 10 min of treatment with peptides and can be blocked by 10 μM amiloride. The time points of peptide and amiloride addition are marked by arrows. The whole-cell current of transiently transfected HEK-293 cells was measured continuously; *n* = 3–5.

Both solnatide and AP318 enhanced amiloride-sensitive Na^+^ current in a concentration dependent manner, although the EC_50_-values of most mutants were higher compared to WT (Table 2). This is most likely due to the changed tertiary structure and subunit assembly of mutant ENaC and therefore a poorer accessibility of the binding sites of the peptides. At 200 nM of solnatide or AP318, a maximum current increase was observed in WT ENaC and in all mutants, which can be seen in the dose-response-curve of solnatide and AP318 of the αC133Y mutant as an example (**Figure 11C**) or in the time-dependent activation curve of WT ENaC with solnatide and AP318 (**Figures 5A,B**). Both peptides restored the current to physiological values of WT control or even higher (Figure 4). Notably, AP318 elicited a higher maximum current compared to solnatide (compare Figures 4A,B) and showed lower EC_50_-values (Table 2) for mutant and WT αβγ-ENaC. The major differences between solnatide and AP318 are the replacement of terminal cysteines of solnatide with 4-aminobutanoic and aspartic acid residues and the consequent absence of a positively-charged N-terminal group in AP318. This difference in chemical structure apparently results in an increased potency of AP318 compared to solnatide.

**Table 2:** **EC_**50**_-values for solnatide and AP318 in WT and mutant ENaC**.

**Construct**	**Solnatide EC_50_ (nM)**	**AP318 EC_50_ (nM)**
Wild type	52.4 ± 3.7	29.3 ± 4.4
αQ101K	64.5 ± 3.5^*^	37.4 ± 1.7
αC133Y	74.0 ± 6.8^***^	42.1 ± 2.4^**^
αS243P	80.1 ± 12.9^***^	49.3 ± 5.8^***^
αG327C	50.2 ± 5.2	26.1 ± 2.9
αS562L	64.3 ± 6.3	46.4 ± 2.9^***^
αS562P	61.1 ± 8.9	49.3 ± 3.1^***^
αS243fs	72.8 ± 1.3^**^	38.9 ± 6.5
βG37S	65.8 ± 3.2^*^	36.7 ± 6.0
γV543fs	58.4 ± 7.9	47.7 ± 4.1^***^

*Mean values ± SEM of 3–8 independent experiments are given. Significant differences compared to WT are indicated*,

* *p < 0.05*,

** *p < 0.01*,

*** *p < 0.001*.

**Figure 6:**
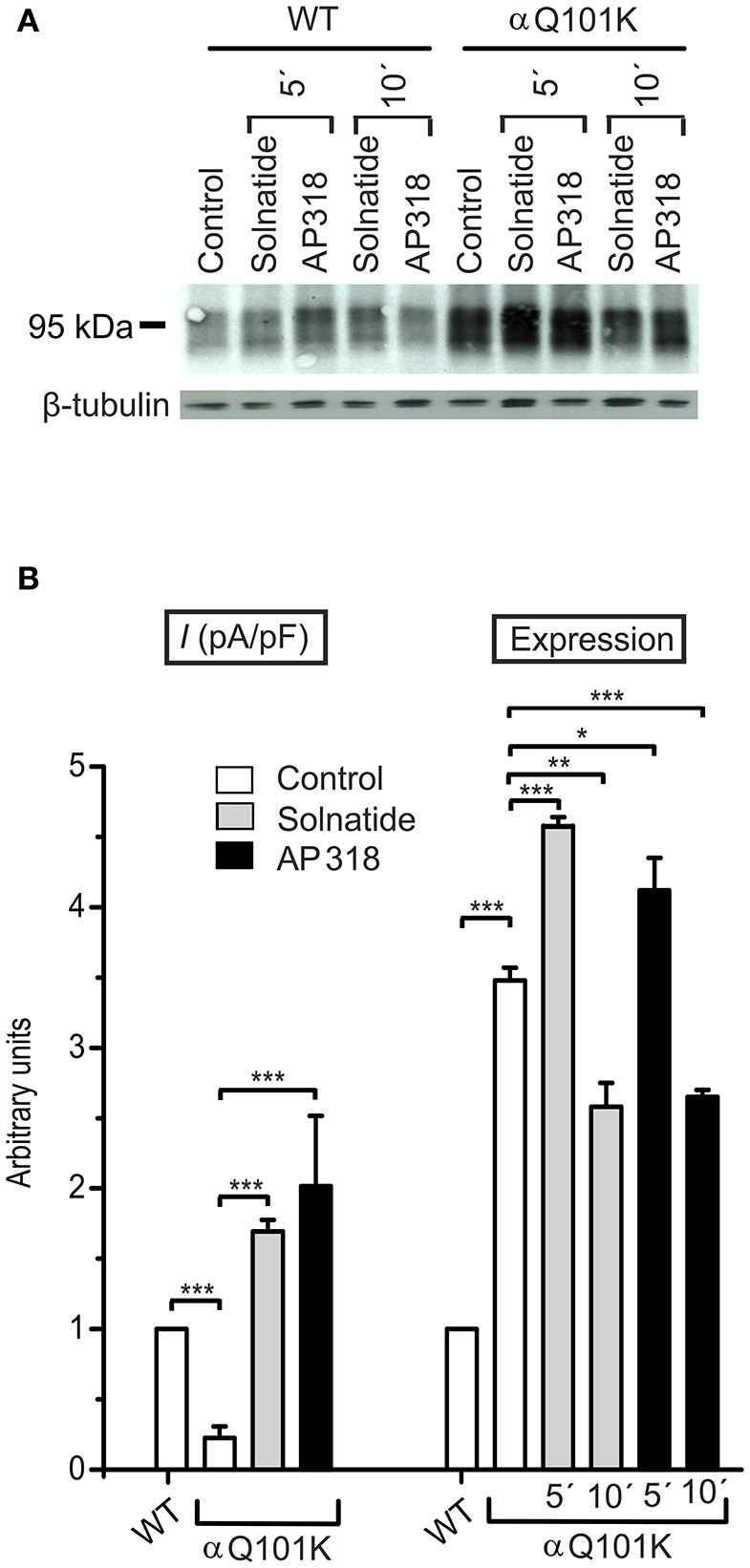
**Both solnatide and AP318 activate macroscopic Na^**+**^ current and increase membrane abundance. (A)** A typical Western blot out of four with biotinylated surface proteins of HEK-293 cells transiently transfected with WT or αQ101K mutant ENaC and treated with solnatide or AP318 at indicated time points is shown, using α-ENaC and β-tubulin antibodies. **(B)** Solnatide- (200 nM, gray bar) and AP318- (200 nM, black bar) induced current in αQ101K was normalized relative to WT control (empty bar). Mean values ± SEM of 6–13 experiments are given. Membrane abundance was increased without (empty bar) and with 200 nM solnatide (gray bars) and AP318 (black bars) at indicated time points (5′ or 10′). Expression of αQ101K ENaC was quantified relative to β-tubulin from Western blots using four independent biological replicates. Significant differences are indicated, ^*^p < 0.05, ^**^p < 0.01, ^***^p < 0.001.

**Figure 7:**
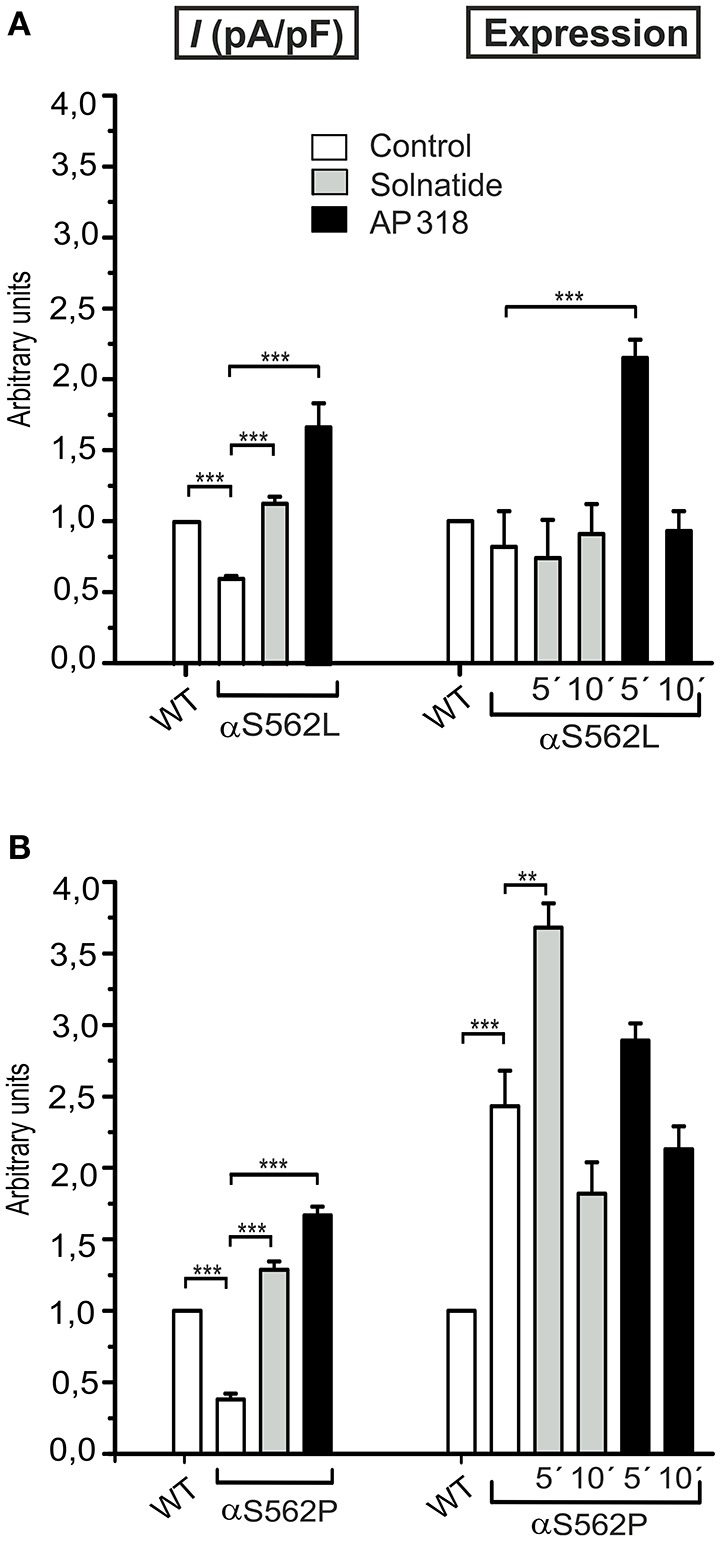
**Solnatide and AP318 show similarity in macroscopic current activation but difference in membrane abundance of ENaC PHA1B mutations at same position**. The macroscopic Na^+^ current (*I* (pA/pF)) of αS562L **(A)** and αS562P **(B)** mutation without (empty bars) and with treatment with 200 nM solnatide (gray bars) and AP318 (black bars) was normalized relative to WT control (empty bars). Both PHA1B mutations at this position of ENaC show a reduced control current compared to WT and treatment with solnatide and AP318 increased the current higher than WT control (*n* = 4–13). The expression of α-ENaC was quantified relative to β-tubulin from Western blots using three independent biological replicates each, and the membrane abundance (expression) of αS562L **(A)** and αS562P **(B)** without or with treatment with solnatide and AP318 at indicated time points was set in relation to WT control. Significant differences are indicated, ^**^p < 0.01, ^***^p < 0.001.

**Figure 8:**
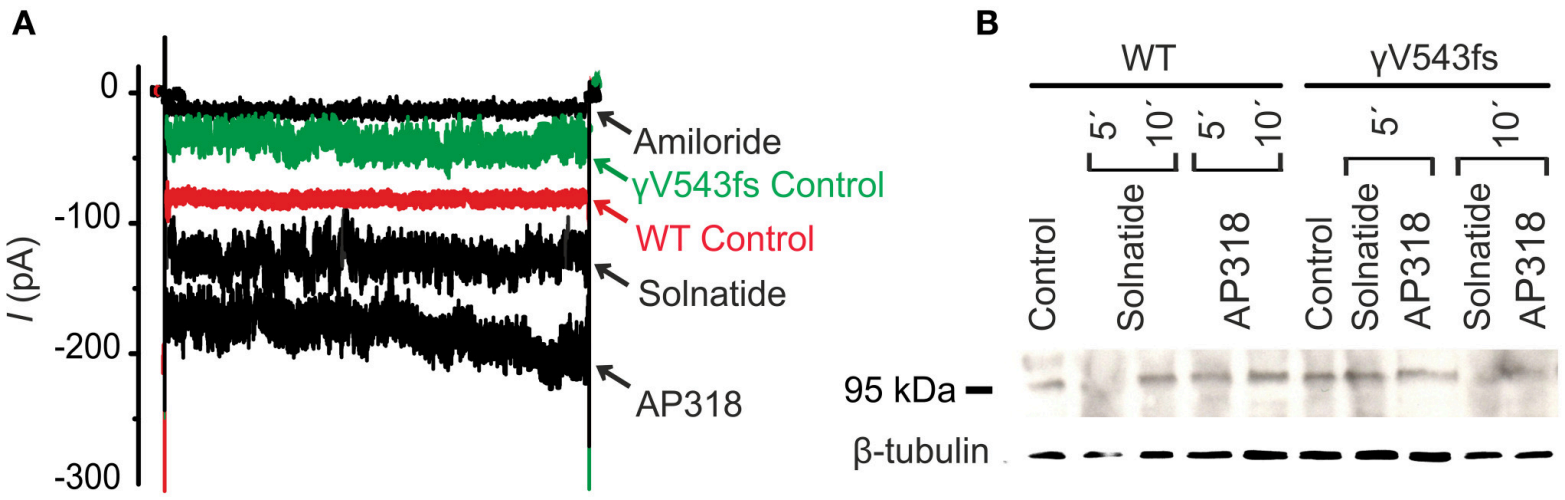
**Solnatide and AP318 activate ENaC currents, but not membrane abundance of γV543fs. (A)** Superimposed representative whole-cell current traces of αβγV543fs ENaC in absence (γV543fs control) and presence of 10 μM amiloride, 200 nM solnatide and 200 nM AP318 are shown as well as WT ENaC control. HEK-293 cells transfected with WT or mutant ENaC channel construct were patched in the whole-cell mode; inward current was elicited at −100 mV (*n* = 3–6). **(B)** Biotinylated proteins from cellular extracts of HEK-293 cells (~15 μg) transiently transfected with WT or PHA1B mutant αβγV543fs ENaC were treated with solnatide and AP318 at indicated time points, and were subjected to SDS/PAGE and immunoblotting analysis with γ-ENaC and β-tubulin antibodies. A typical blot out of five independent biological replications is shown.

**Figure 9:**
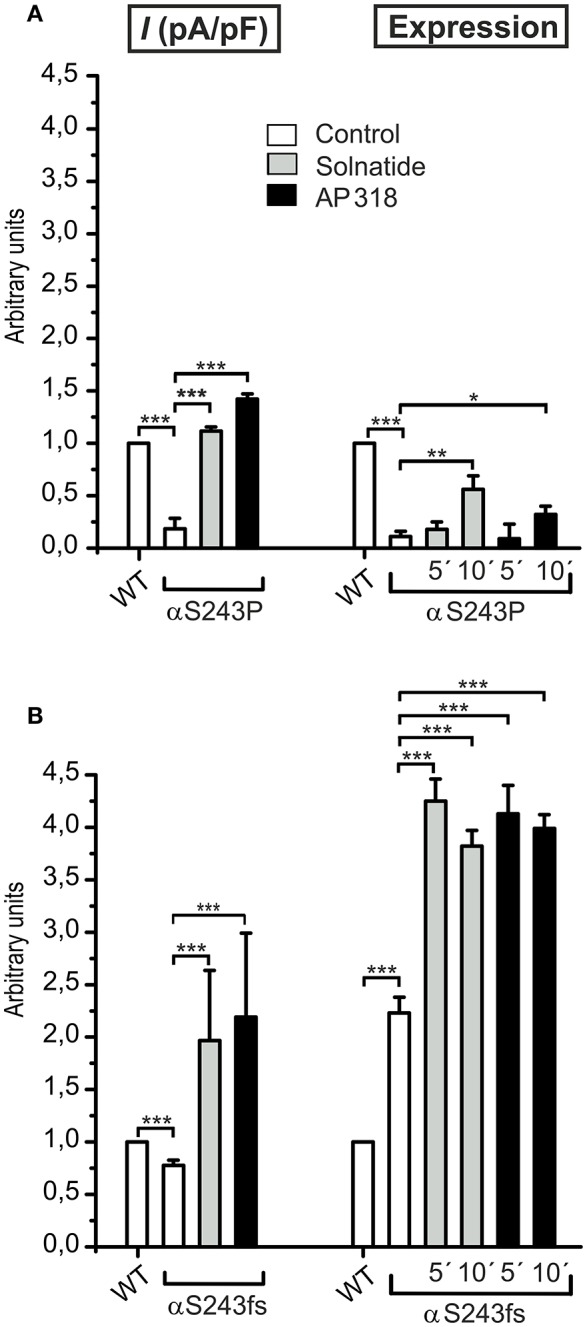
**Point and frameshift mutation show different current activation and membrane abundance after treatment with solnatide and AP318**. The macroscopic Na^+^ current (*I* (pA/pF)) of αS243P **(A)** and αS243fs **(B)** PHA1B mutation without (empty bars) and with treatment with 200 nM solnatide (gray bars) and AP318 (black bars) was normalized relative to WT control (empty bars) (*n* = 3–13). The control currents of both mutations at the same position of ENaC are reduced compared to WT and can be rescued by solnatide and AP318. The expression of α-ENaC was quantified relative to β-tubulin from Western blots using four independent biological replicates each, and the membrane abundance (expression) of αS243P **(A)** and αS243fs **(B)** without or with treatment with solnatide and AP318 at indicated time points was set in relation to WT control. The surface expression of αS243P is reduced and only improved after 10 min treatment with solnatide and AP318, whereas the membrane abundance of αS243fs is more than double compared to WT control and further enhanced by solnatide and AP318 treatment. Significant differences are indicated, ^*^p < 0.05, ^**^p < 0.01, ^***^p < 0.001.

**Figure 10:**
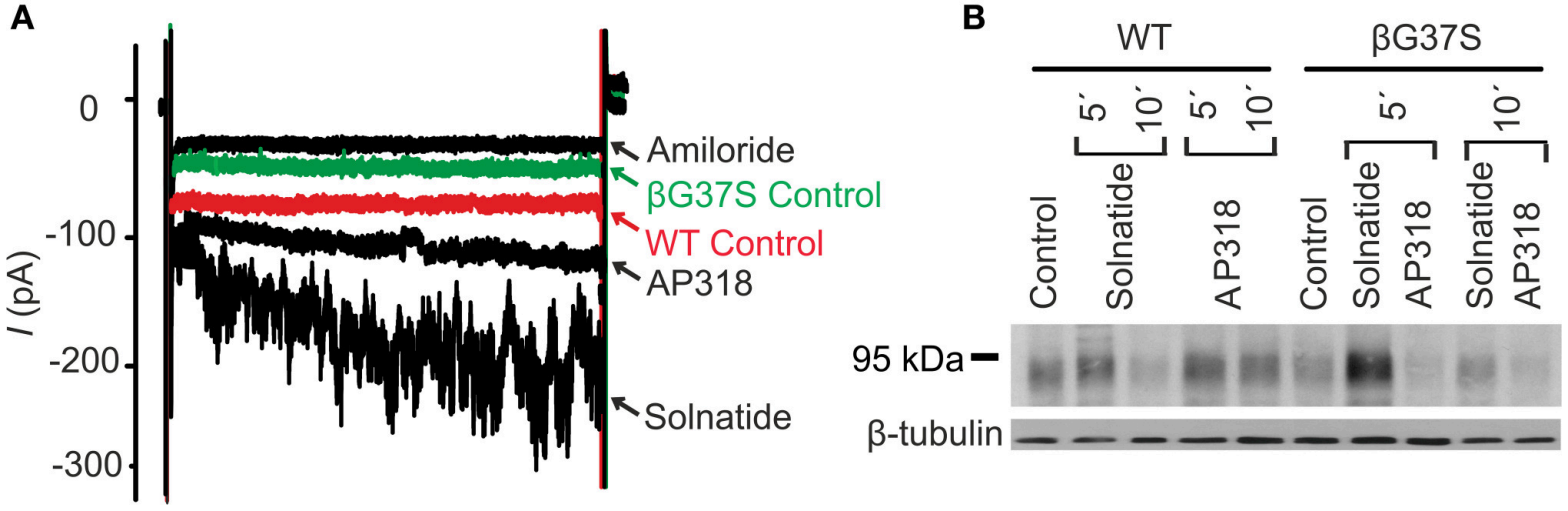
**Both solnatide and AP318 activate ENaC currents, but only solnatide increases membrane abundance in βG37S. (A)** Superimposed representative whole-cell current traces of αβG37Sγ ENaC in absence (βG37S control) and presence of 10 μM amiloride, 200 nM solnatide and 200 nM AP318 are shown as well as WT ENaC control. The HEK-293 cells transfected with indicated ENaC channel construct were patched in the whole-cell mode; inward current was elicited at −100 mV (*n* = 5–13). **(B)** Biotinylated proteins from cellular extracts of HEK-293 cells (~15 μg) transiently transfected with WT or PHA1B mutant αβG37Sγ ENaC were treated with maximum current activating solnatide and AP318 concentrations at indicated time points, and were subjected to SDS/PAGE and immunoblotting analysis with β-ENaC and β-tubulin antibodies. A typical blot out of five independent biological replications is shown.

**Figure 11:**
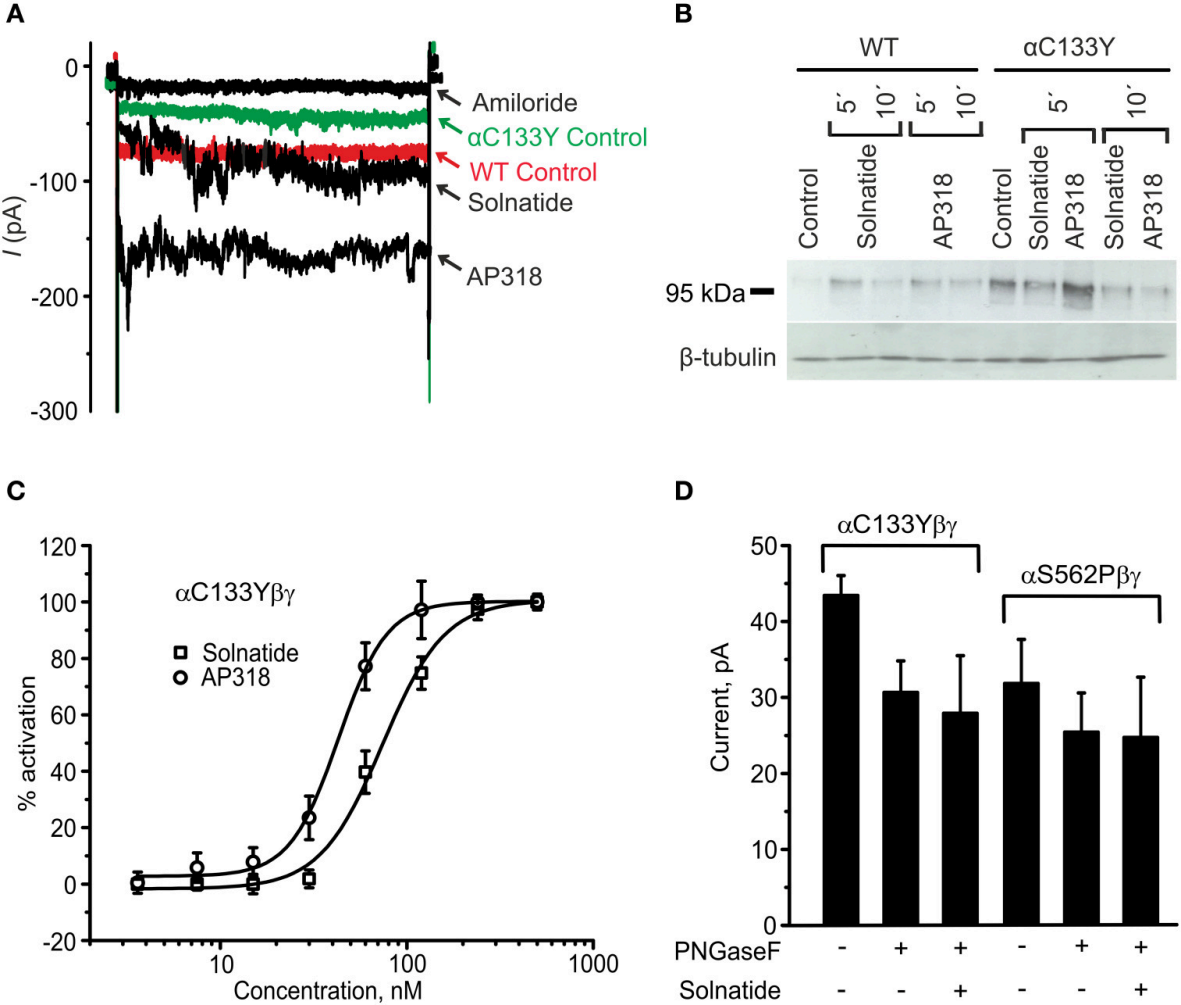
**Original recordings of patch-clamp and Western blot experiments, dose-response-curve of solnatide and AP318 and the effect of deglycosylation on the current activation of αC133Y. (A)** Superimposed representative whole-cell current traces of αC133Yβγ ENaC in absence (αC133Y control) and presence of 10 μM amiloride, 200 nM solnatide and 200 nM AP318 are shown as well as WT ENaC control. HEK-293 cells transfected with WT or mutant ENaC channel construct were patched in the whole-cell mode; inward current was elicited at −100 mV (*n* = 3–13). **(B)** Biotinylated proteins from cellular extracts of HEK-293 cells (~15 μg) transiently transfected with WT or PHA1B mutant αC133Yβγ ENaC were treated with maximum current activating solnatide and AP318 concentrations at indicated time points, and were subjected to SDS/PAGE and immunoblotting analysis with α-ENaC and β-tubulin antibodies. A typical blot out of five independent biological replications is shown. **(C)** A dose-response-curve of solnatide and AP318 was obtained by cumulatively adding one of the peptides into the bath solution during patch-clamp experiments. Solnatide: *n* = 5; AP318: *n* = 3. **(D)** After treatment with PNGase F, which deglycosylates ENaC, no effect of solnatide can be observed in whole-cell patch-clamp experiments with αC133Y and αS562P mutants, *n* = 3.

**Figure 12:**
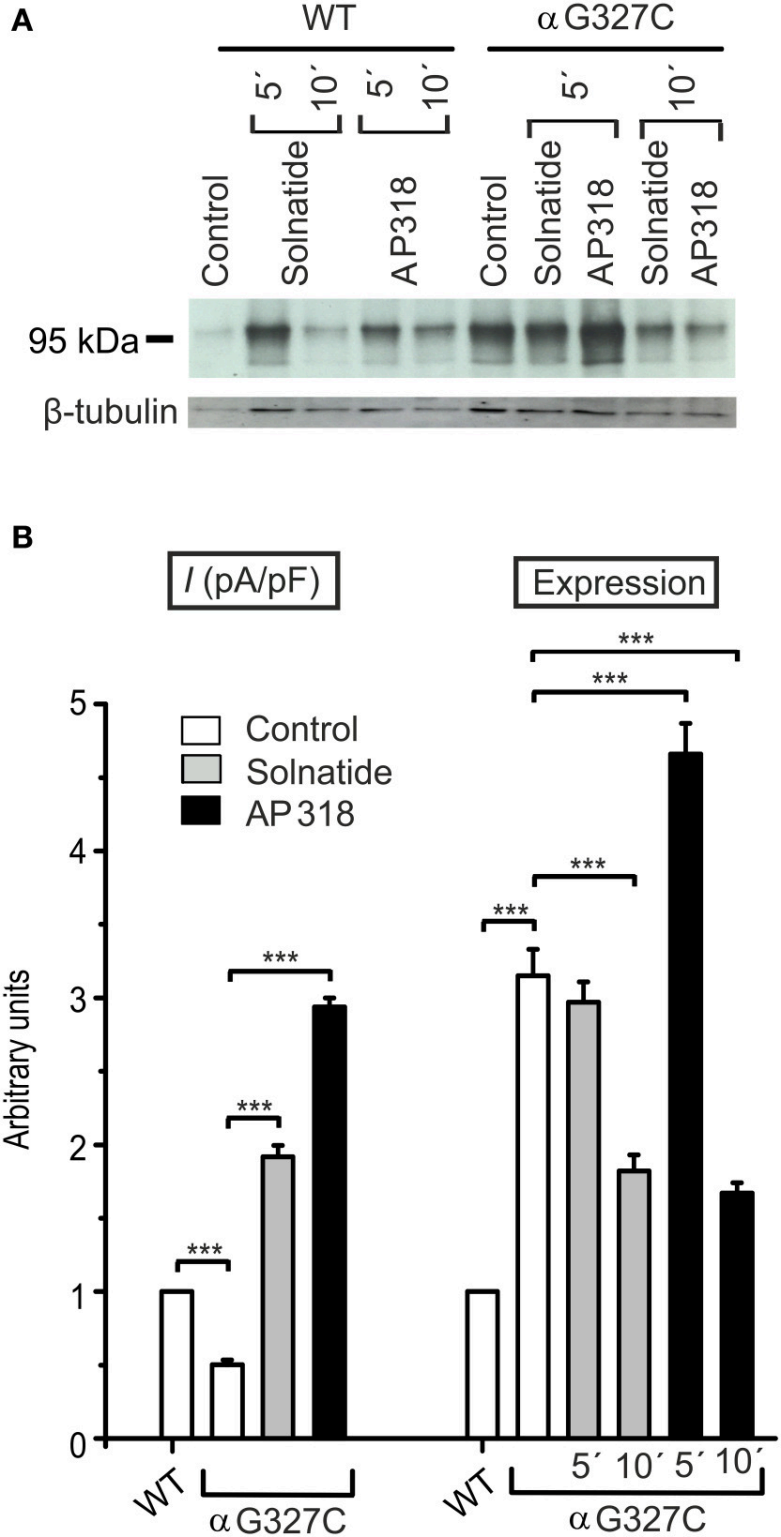
**Both solnatide and AP318 activate macroscopic Na^**+**^ current, but only AP318 increases membrane abundance. (A)** A typical Western blot out of three with biotinylated surface proteins of HEK-293 cells transiently transfected with WT or αG327C mutant ENaC and treated with solnatide or AP318 at indicated time points is shown, using α-ENaC and β-tubulin antibodies. **(B)** The current of αG327C without (empty bar) and with treatment with 200 nM solnatide (gray bar) and AP318 (black bar) was normalized relative to WT control (empty bar) (*n* = 4–13). The control current is reduced compared to WT and can be rescued by solnatide and AP318. Membrane abundance was increased without (empty bar) and with 200 nM solnatide (gray bars) and AP318 (black bars) at indicated time points (5′ or 10′) compared to WT control. The expression of α-ENaC was quantified relative to β-tubulin from Western blots using three independent biological replicates. Significant differences are indicated, ^***^p < 0.001.

For further investigation the macroscopic Na^+^ current and membrane abundance of ENaC carrying PHA1B mutations before and after treatment with solnatide and AP318 were set in relation to WT control (Figures 6, 7, **9**, **12**). Original trace recordings are shown for one mutation in each ENaC subunit (Figures 8, **10**, **11**). Additionally an example of a dose-response-curve of solnatide and AP318 of the αC133Y mutant was included into **Figure 11C**.

Since the glycosylation sites of α-ENaC have been shown to be essential for the activating effect of solnatide (Shabbir et al., 2013, 2015), it was tested, whether this is still the case for PHA1B mutant ENaC. Therefore, the effect of solnatide was tested exemplarily on some mutants after treatment with PNGase F, which deglycosylates the cell membrane of the transfected HEK-293 cells. Solnatide had no effect on these mutant ENaCs after deglycosylation (**Figure 11D**), similar to the results observed previously for WT ENaC (Shabbir et al., 2013).

Mutations are discussed below according to three themes: (1) effect of mutations in the two TM regions of ENaC, namely αQ101K, αS562L, αS562P, and γV543fs; (2) effect of frameshift mutations compared to point mutations; and (3) effect of mutations in non-TM regions, namely αC133Y, αG327C, and βG37S.

### PHA1B mutations in TM regions

ENaC carrying the αQ101K and γV543fs mutations showed a very low control current indicating severe loss-of-function; αS562L and αS562P, on the other hand showed about half of the WT control current, a moderate decrease compared to the other mutations (Figure 3). As mentioned above solnatide and AP318 were able to rescue all the mutants. The fold increase of current in the αQ101K and γV543fs mutations are about 8-fold for solnatide and 9-fold for AP318, similar to WT ENaC (10-fold increase with solnatide and 11-fold with AP318; compare Shabbir et al., 2013). The αS562L mutant shows an approximately 2-fold increase with solnatide and 3-fold with AP318, and αS562P 4-fold with solnatide and 5-fold with AP318. Remarkably, the cell surface expression of αQ101K was far higher than WT control and even increased after 5 min treatment with solnatide and AP318 (Figure 6). After 10 min treatment with both peptides the surface expression of αQ101K is decreased, but still higher than WT. The whole-cell current is rather unaffected by the changed membrane abundance and is consistently increased at least for 10 min after treatment with solnatide (Figure 5C). The αS562P mutation also showed increased membrane abundance in control and after short-term exposure to solnatide and AP318. In contrast the expression of αS562L remained unchanged compared to WT, with membrane abundance increasing only after 5 min in the presence of AP318 (Figure 7). For comparison, the surface expression of WT α-ENaC showed a 1.5-fold increase after 5 min treatment with solnatide and 1.4-fold with AP318, when co-expressed with WT βγ-subunits (see Table 3). The surface expression of γV543fs—but also of WT γ-ENaC (see also Shabbir et al., 2015)—did not increase significantly, when set in relation to respective β-tubulin quantity (Figure 8).

**Table 3:** **Membrane abundance of WT and mutant ENaC without and with 5 and 10 min treatment with solnatide and AP318**.

**Mutation**	**Control**	**Solnatide**		**AP318**	
		**5 min**	**10 min**	**5 min**	**10 min**
Wild type	1	1.46 ± 0.12^**^	1.34 ± 0.23	1.41 ± 0.13^**^	1.38 ± 0.16^*^
αQ101K	3.48 ± 0.09^†††^	4.58 ± 0.06^***^	2.58 ± 0.17^**^	4.12 ± 0.23^*^	2.65 ± 0.05^***^
αC133Y	2.25 ± 0.14^†††^	1.89 ± 0.04^*^	1.51 ± 0.08^**^	3.84 ± 0.16^***^	1.28 ± 0.05^***^
αS243P	0.11 ± 0.05^†††^	0.18 ± 0.07	0.56 ± 0.13^**^	0.09 ± 0.14	0.32 ± 0.08^*^
αG327C	3.15 ± 0.18^†††^	2.97 ± 0.14	1.82 ± 0.11^***^	4.66 ± 0.21^***^	1.67 ± 0.07^***^
αS562L	0.82 ± 0.25	0.74 ± 0.27	0.91 ± 0.21	2.15 ± 0.13^***^	0.93 ± 0.14
αS562P	2.43 ± 0.25^†††^	3.68 ± 0.17^**^	1.82 ± 0.22	2.89 ± 0.12	2.13 ± 0.16
αS243fs	2.23 ± 0.15^†††^	4.25 ± 0.21^***^	3.82 ± 0.15^***^	4.13 ± 0.27^***^	3.99 ± 0.13^***^
βG37S	0.86 ± 0.15	3.47 ± 0.26^***^	0.84 ± 0.15	0.74 ± 0.26	0.56 ± 0.08
γV543fs	1.11 ± 0.15	1.32 ± 0.19	0.44 ± 0.12^**^	1.41 ± 0.06	0.53 ± 0.14^*^

*The amount of α-, β- or γ-ENaC in biotinylated surface proteins of HEK-293 cells transiently transfected with WT or mutant ENaC constructs was quantified by Western blot experiments according to β-tubulin. All samples were set in relation to the α- (see Wild Type), β- or γ- (data not shown; compare Shabbir et al., 2015) ENaC surface expression of WT control, respectively. For mutant ENaC mean values ± SEM of 3–5 independent biological replicates are given (WT: n = 26). All controls were compared to WT control*,

††† *p < 0.001. After treatment with solnatide and AP318 at indicated time points significant difference was calculated compared to the control values of the respectively same mutation*,

* *p < 0.05*,

** *p < 0.01*,

*** *p < 0.001*.

### Effect of solnatide and AP318 on point vs. frameshift mutations

The ENaC mutant αS243P showed a very low control current, whereas the macroscopic Na^+^ current of the frameshift mutation at the same position was only slightly reduced compared to WT. The current activation by solnatide and AP318 looked similar for both mutations, when compared to the respective control current. The fold activation for both mutations and peptides is around 3-fold. While the αS243P mutation showed a very low surface expression, which was only slightly, but significantly increased after 10 min by solnatide and AP318 and did not reach the WT value, the αS243fs mutation had an increased membrane abundance without and even higher with solnatide and AP318 (Figure 9). The control surface expression of αS243fs was even higher than WT α-ENaC after treatment with solnatide and AP318 (Table 3). Worth mentioning in Western blot of biotinylated surface proteins from HEK-293 cells transiently transfected with αS243fsβγ-ENaC a band was observed at about 30 kDa, which corresponds with the expected size 28.6 kDa of the truncated α-ENaC (theoretical protein length: 247 amino acids).

### Non-TM PHA1B mutations

The control current of the βG37S mutant was low, but increased after treatment with solnatide and AP318. The fold increase with solnatide is about 7-fold and with AP318 5-fold. The surface expression of βG37S showed no appreciable change compared to WT, but after 5 min treatment with solnatide a high increase was observed (Figure 10), which is particularly interesting since in previous studies the surface expression of WT β-ENaC was not affected by solnatide (Shabbir et al., 2015). The macroscopic Na^+^ current of αC133Y was reduced compared to WT control and could be rescued by solnatide and AP318, the fold increase for solnatide being about 2-fold and for AP318 3.3-fold. The membrane abundance of αC133Y was higher than WT control and even higher than WT after treatment with solnatide and AP318 (Table 3), and increased after 5 min of AP318 treatment (Figure 11). The control current of αG327C was decreased as in all tested PHA1B mutations, but it showed the highest increase in current among all investigated mutations after treatment with AP318 (Figure 4). Also among all tested mutations it was the only one, on which both peptides did not change their EC_50_-values compared to the WT channel (Table 2). Nevertheless, the fold increase around 6-fold for both peptides is lower than the 10-fold increase of WT current after treatment with solnatide and 11-fold with AP318. The membrane abundance was highly increased and further increased upon treatment with AP318 after 5 min (Figure 12). After 10 min treatment with both peptides the cell surface expression of αG327C decreased, but is still higher than WT α-ENaC. This time-dependent change of expression does not influence the measured whole-cell current, which is consistently increased at least for 10 min after treatment with AP318 (Figure 5D).

In summary, the macroscopic Na^+^ currents of all investigated conserved PHA1B mutants were decreased, and could be activated by treatment with solnatide or AP318 up to or even higher than the WT control level. A greater variation among the mutations was observed regarding their membrane abundance. The surface expression of some mutants like αS243P was sharply reduced, while the membrane abundance of others, like αQ101K was markedly increased. While the surface expression of WT channel was increased approximately 1.4-fold by solnatide and AP318, the peptides apparently had no effect on membrane abundance of some PHA1B mutants but a greater effect compared to WT on others. For the most mutants of α-ENaC (e.g., αQ101K and αG327C) the membrane abundance is decreased after 10 min of solnatide and AP318 treatment (Table 3). A similar effect was reported for WT ENaC by Shabbir et al. (2015), where the highest surface expression of α-ENaC was observed after 5 min of solnatide treatment and after 10 and 30 min the expression decreases again, but is still higher than control. The effect of solnatide and AP318 on the cell surface expression of WT or mutant ENaC is very rapid and transient; therefore it is unlikely that the observed effect is due to transcription of ENaC genes. More likely ENaC channels are translocated from the Golgi apparatus to the cell surface membrane or the degradation of ENaC mediated by Nedd4-2 is inhibited. Remarkably, the observed solnatide- and AP318-induced current was not (only) caused by increased membrane abundance of ENaC channels, but the peptides additionally increase the open probability, *P*_*o*_ of ENaC persistently, which has already been shown for WT ENaC channels (Shabbir et al., 2013, 2015). These two effects seem to occur by different mechanisms, because the peptides affect the membrane abundance of ENaC only for a short time of 5 or 10 min; at longer exposure times the values return to control; whereas the ENaC-current is increased persistently. As shown by Lucas et al. (2016), the open probability of WT ENaC transiently transfected in H441 cells is still increased 15 min after addition of solnatide (TIP peptide). In primary alveolar epithelial type II cells and additionally in A549 cells, endogenously expressing ENaC, solnatide was able to increase the whole-cell current consistently for about 15 min (Tzotzos et al., 2013). Although some PHA1B mutants showed a different—for some mutants even stronger—time-dependent effect of solnatide and AP318 on the membrane abundance compared to WT ENaC, the peptide-induced increase in current was still persistent at least for 10 min of peptide treatment, as exemplarily shown for αQ101K (Figure 5C) and αG327C (Figure 5D). Secondly, some mutations do not show an increase in membrane abundance after treatment with solnatide and AP318, but currents of all mutant ENaC constructs are activated by the peptides.

## Discussion

The widespread tissue distribution of ion channels and the diversity of their physiological functions, gives rise to a range of channelopathies caused by mutations in genes encoding ion channel subunits, or their interacting proteins. For most channelopathies described to date, therapy is mainly empirical and symptomatic, often limited by lack of efficacy and tolerability. Nevertheless, despite the potential of ion channels as drug targets, relatively few marketed drugs have ion channels as their primary target. PHA1B is a rare, life-threatening, inherited, salt-wasting disease which is associated with mutations in ENaC resulting in a decrease of channel activity. Previously, solnatide was shown to increase ENaC activity in A549 and H441 cells (Hazemi et al., 2010; Shabbir et al., 2013; Czikora et al., 2014), freshly isolated type II alveolar epithelial cells from different species (Tzotzos et al., 2013) and heterologously expressed human ENaC subunits (Shabbir et al., 2013, 2015). A similar pronounced increase in ENaC activity with an even smaller EC_50_-value was observed with AP318 in A549 cells (Hazemi et al., 2010; Shabbir et al., 2013). Hence we intended to prove whether this ENaC-activating effect applies to mutated ENaC which causes PHA1B. It was demonstrated that αβγ- (Canessa et al., 1994; Edelheit et al., 2011) or δβγ-ENaC subunit coexpression (Shabbir et al., 2013) was essential for maximal activity in control as well as treatment with solnatide (Shabbir et al., 2013). Thus, in our experiments we always co-expressed the specific mutated subunit together with the two appropriate WT subunits, to achieve heterooligomeric ENaC expression.

### Effect of TIP peptides on ENaC carrying mutations in TM regions

According to homology with chicken ASIC1 (Jasti et al., 2007), the point mutations αQ101K, αS562L, αS562P are situated in the transmembrane regions TM1 (F86-F106) and TM2 (M544-L577, equivalent to V427-I460 in ASIC1) of α-hENaC. AlphaQ101K lies within TM1, five residues from the extracellular surface of the membrane, whereas the mutations αS562L and αS562P occur in the lower middle of TM2.

In our experiments amiloride-sensitive Na^+^ current was significantly lower in mutants situated in or near the transmembrane regions compared to WT ENaC. Our findings thus confirm those of Boiko et al. (2015) for the αS562P mutant who recorded almost no current in this mutant. In the case of αQ101K, αS562L, and αS562P mutants, the two test compounds, solnatide and AP318, were able to increase the amiloride-sensitive Na^+^ current to WT control level or even above, AP318 showing even higher activity than solnatide. However, membrane abundance differed markedly among these mutants. While membrane expression of αQ101K and αS562P was significantly increased in control, expression of αS562L was almost unaffected. Both TIP peptides caused a transient increase in membrane abundance of αQ101K and αS562P, but not of the αS562L mutant.

Q101 is part of the highly conserved “YWQF” motif, and sequence alignment shows that the “YWQF” motif is conserved in different ENaC subunits and species (Mora-Lopez et al., 2012). In the case of NMDA receptors it could be demonstrated that the “YWQF” motif is necessary and sufficient to drive their internalization (Scott et al., 2004; Lau and Zukin, 2007). This motif likely plays a similar role in other ion channels. So, it might also be involved in driving ENaC to internalization and degradation in endosomes. According to this hypothesis, disruption of the “YWQF” motif would result in accumulation of ENaC subunits in the membrane, which could explain our findings of increased membrane abundance of mutant α-subunit in the case of the αQ101K mutant.

Previous studies have shown that selected mutations within the TM2 tract have dramatic effects on channel gating (Waldmann et al., 1995; Snyder et al., 2000). Mutagenesis studies support the concept that the selectivity filter of ENaC involves three conserved amino acids, G/SxS in the middle of the TM2 segment in α-, β-, and γ-subunits. S562 is the third residue of the “GSS” selectivity motif described by Kellenberger et al. (2001). The authors reported that mutation of S589 in rat α-ENaC, (equivalent to S562 in human α-ENaC), caused loss of ENaC function. So, the *in vitro* study reflected the *in vivo* report of the human mutation which causes PHA1B (Schaedel et al., 1999). The mutation S562P was found in a Somalian family with the typical systemic PHA1B phenotype. The role of residues homologous to αS562 for the ionic selectivity of other ENaC/DEG channels such as DEG-1 (Garcia-Anoveros et al., 1995), MEC-4 (Hong and Driscoll, 1994), and ASIC2a (Waldmann et al., 1996) clearly points to their critical roles in ENaC function. Together with the corresponding residues in the β- and γ-ENaC subunits, S562 forms the ENaC selectivity filter (Kellenberger et al., 1999a,b), lining the channel pore at its narrowest part. Our findings for the αS562P mutation are consistent with published data which revealed a dramatic loss-of-function of the mutant in *in vitro* expression (Boiko et al., 2015). It is assumed that reduced ion conduction is likely due to structural changes in the critical region of the selectivity filter. Contrary to Riepe (2009) who found similar membrane expression of this mutant, we observed a significant increase in expression of αS562P compared to WT ENaC, whereas membrane abundance was not significantly changed in the αS562L mutant. This discrepancy between these two mutants at position 562 regarding membrane abundance might be due to physico-chemical properties of the amino acids. Riepe (2009) reported that artificial mutation of S562 changing serine to amino acids of similar sizes make ENaC permeable for larger ions, whereas substitution of S562 with aromatic residues inactivates the channel. Both substituted amino acids, proline in the S562P and leucine in the S562L mutants, are non-polar and neutral, but differ in hydrophobicity and van-der-Waals volume. The substitution of the polar residue serine for the non-polar proline would introduce rigidity into the polypeptide chain and render it inflexible, whereas the introduction of leucine with its bulky hydrophobic side chain would bring about steric hindrance compared to the smaller, polar side chain of the WT serine. Both TIP peptides increased the reduced current in αS562L as well as αS562P above WT ENaC levels, but whereas both TIP peptides transiently enhanced membrane abundance in the αS562P mutant, in the αS562L mutant only AP318 caused a transient increase of membrane expression. Solnatide has been shown to activate ENaC upon binding to a domain between the TM2 and the carboxyl terminus of the α-subunit of the channel (Czikora et al., 2014; Lucas et al., 2016), and apparently this binding was not prevented by these mutations located in TM2, so that current could be restored to WT levels or above.

For γ-hENaC, sequence alignment with chicken ASIC1 specifies the residues I524-E551 for TM2, because after E551 (equivalent to D454 in chicken ASIC1) significant sequence similarity between chicken ASIC1 and γ-hENaC is not detectable. According to this interpretation, the frameshift mutation γV543fs lies at the lower part of TM2 eight residues away from the intracellular surface of the membrane, which is also within the TM2 range of the model published by Stockand et al. (2008). Interestingly residue S542, preceding V543 in γ-ENaC, is the equivalent residue to S562 in α-ENaC, which is the third residue of the selectivity filter “GSS” motif in the α-subunit as discussed above. Thus, V543 lies adjacent to the “selectivity filter” in γ-hENaC, which could explain why γV543fs affects ENaC function. Indeed, in control conditions current was markedly decreased compared to WT ENaC, whereas membrane abundance remained almost unchanged. Nonetheless, in the γV543fs mutant amiloride-sensitive current and membrane abundance were significantly increased by both test compounds. Thus, it can be assumed that after binding to glycosylation sites (Shabbir et al., 2015) solnatide (Lucas et al., 2016) and AP318 still can interact with the C-terminal domain of the α-subunit in the γV543fs mutant ENaC heterooligomer and increase current in this way, although to a lesser extent than in WT ENaC. This confirms our findings in WT ENaC which showed that solnatide activates current in the dual αβ-ENaC subunit combination to a similar extent (Shabbir et al., 2013) as observed in γV543fs co-expressed with αβ-ENaC subunits.

### Effect of TIP peptides on point vs. frameshift mutation

Point mutation αS243P and frameshift mutant αS243fs are located within the finger domain of ENaC. The finger domain shows the least sequence conservation within the ENaC/DEG family (Jasti et al., 2007). This sequence variability is believed to reflect the ability of members of the ENaC/DEG family of ion channels to respond to a variety of extracellular signals which affect channel opening or closing. In this sense the finger domains of these proteins may be viewed as functional modules. In the case of α-ENaC, the finger domain has been associated with activation of ENaC through protease cleavage (Kashlan et al., 2010, 2011), Na^+^ sensitivity and Na^+^ self-inhibition (Kashlan et al., 2010), as well as the modulation of channel activity in response to shear stress (Shi et al., 2012).

Both mutants, αS243P and αS243fs, showed a marked decrease in amiloride-sensitive current, which was even more pronounced in the case of the point mutation. Similar to the αS562P mutant (Boiko et al., 2015) discussed above, a change of the polar amino acid serine at position 243 to the heterocyclic, non-polar proline would introduce rigidity into the protein backbone which might in turn result in a deleterious change to the tertiary structure of the protein, thereby interfering with function. In addition, the mutants prominently differed regarding membrane expression. Whilst membrane abundance of αS243P was dramatically reduced, the frameshift mutant was significantly more highly expressed in the membrane compared to WT ENaC. Residue S243 is located fifty or so amino acids further along the polypeptide chain from the furin cleavage sites flanking the inhibitory tract between R178 and R204, the excision of which is required for full ENaC activity due to suppression of Na^+^ self-inhibition (Kleyman et al., 2009; Kashlan et al., 2010, 2012; Hanukoglu and Hanukoglu, 2016), so it is unlikely that the mutated residue would interfere with the cleavage reaction. However, in the homology model of mouse α-ENaC, S270, the equivalent residue to S243 in human α-ENaC lies in proximity with the central portion of the inhibitory peptide (Kashlan et al., 2011). It is likely that the marked decrease in amiloride-sensitive current observed with the S243P mutant is due to this mutation interfering with release of the cleaved peptide from the channel surface, thus favoring self-inhibition. The substituted proline residue introduces rigidity into the protein backbone and the resulting conformational inflexibility could prevent release of the inhibitory peptide. Regarding the S243fs mutation a truncated protein of 247 residues with a bulky C-terminus (S242SGWMR-COOH) is produced, which may not be correctly folded, either for furin cleavage or for heterooligomeric assembly. These results are consistent with several studies in which systematic mutagenesis of residues in this region of α-ENaC has demonstrated their involvement in Na^+^ self-inhibition (Kashlan et al., 2010, 2011). Moreover, the S243fs mutant lacks the “PPxY” and “YXXΦ” motifs located in the intracellular carboxyl terminal region and required for ubiquitination and endocytosis, in the absence of which subunits accumulate at the cell surface (Wiemuth et al., 2007; Bobby et al., 2013). This would explain the significantly higher expression in the membrane of the frameshift mutant compared both to WT ENaC as well as to the S243P point mutant.

The extracellular loop of all ENaC subunits contains 16 conserved Cys residues that can be grouped into two cysteine-rich domains (CRDs). One pair of cysteines, (C229 and C236 in human α-ENaC) in the finger domain is not present in ASIC1 and so the disposition of these cysteines is so far unclear (Sheng et al., 2000). Chemical cross-linking to the finger domain cysteines of both the α- and γ-subunits has revealed that they lie in close proximity to the finger-thumb domain interface of the respective subunits; furthermore this cross-linking affected ENaC currents, suggesting mechanical linkage between this peripheral site and the channel gate (Blobner and Kashlan, 2016).

Both whole-cell current and subunit membrane abundance, were significantly increased by solnatide and AP318. The TM2 domain of the glycosylated α-subunit has recently been identified as a crucial target for binding of TIP peptides (Czikora et al., 2014; Shabbir et al., 2015). So frameshift mutations which precede the C-terminal domain will disrupt this condition for drug binding, as is the case for the frameshift at position 243 which results in a premature stop codon only four amino acids after the affected serine residue, and hence a truncated polypeptide chain. Moreover, we have previously shown that solnatide (Shabbir et al., 2013) and AP318 (unpublished data) can induce an increase in amiloride-sensitive Na^+^ current with βγ-ENaC, whereas the membrane abundance of β- and γ-ENaC stays unchanged (Shabbir et al., 2015). Interestingly the amiloride-sensitive control current and solnatide-induced current of αS243fs co-expressed with βγ-ENaC were significantly higher than βγ-ENaC alone (compare with Shabbir et al., 2013), indicating that even the truncated α-subunit retains some kind of functionality or at least stabilizes the channel complex. This implies that the TIP peptides are able to activate ENaC via an additional mechanism, although this mechanism appears to be of secondary importance under normal conditions.

### Effect of TIP peptides on non-TM mutants

Both peptides, solnatide and AP318, increased reduced amiloride-sensitive current in αC133Y, αG327C and βG37S mutants to control levels of WT ENaC or even higher. αC133Y occurs in the first cysteine rich domain of ENaC (Firsov et al., 1998; Bonny et al., 1999, 2002; Bonny and Hummler, 2000). Mutations in this region might influence the formation of disulphide bonds and consequently tertiary structure of the channel protein, however, obviously without preventing binding of the TIP peptides to the postulated binding sites. Edelheit et al. (2011) showed that mutation of specific conserved charged residues to alanine may affect the surface density of α-ENaC, and that there was a high degree of correlation between ENaC Na^+^ conductance activity and the surface density of ENaC. We could not find this correlation for αC133Y. On the contrary, for αC133Y we found significantly higher membrane expression. So, there was a clear discrepancy between functionality and membrane abundance for this mutant. The αC133Y mutation would disrupt the disulfide bridge between C133 and C305 (Sheng et al., 2007) located on the neighboring strand in the β-ball (β5 according to the homology model of mouse α-ENaC) (Kashlan and Kleyman, 2011), and therefore important for the structural integrity of this domain which lies at the core of the subunit. It is likely that this mutation causes serious disruption to the conformation of the subunit.

In our setting, current reduction in the αG327C mutant (Edelheit et al., 2005) is consistent with the data published by Hanukoglu et al. (2008). These authors suggested that the reason for reduced ENaC activity in the mutant could be due to the larger side chain of cysteine compared to the more flexible glycine which would weaken or interfere with subunit interaction (Hanukoglu et al., 2008). Furthermore, the cysteine of the mutant could potentially participate in formation of non-native disulphide bonds during the folding process resulting in a misfolded subunit. According to alignment with the Kashlan model of α-mENaC (Kashlan and Kleyman, 2011), the mutation αG327C is located on the surface of the α-subunit at the boundary between the β-ball and palm domains and near the vertical axis of rotational symmetry down the center of the trimer. Edelheit et al. (2014) suggested it is likely that residues at this location are involved in conformational changes that lead to channel constriction and the Na^+^ self-inhibition response upon Na^+^ ion flooding. The αG327C mutant, of all mutants described in this work, is the one which showed the largest response to activation of Na^+^ current by both peptides—particularly so to AP318. Considering the putative location of this mutation at the interface of the three subunits, this observation lends weight to the argument that TIP peptides exert their effect by interfering with intersubunit conformational changes.

The mutant βG37S occurs in the HG motif of ENaC channels. ENaC carrying mutations in the HG motif show altered gating properties characterized by abnormally long closures and short channel openings corresponding to a loss-of-function mutation (Chang et al., 1996; Gründer et al., 1997). Reduced whole-cell current was accompanied by slightly lower membrane expression, but access of the TIP peptides to the binding site in the α-subunit is apparently not inhibited by the mutation in the N-terminal domain of the β-subunit.

## Conclusion

There are different proven or speculative reasons for reduced functionality of the ion channel, resulting in altered channel pore formation and gating as well as protein expression in the membrane. However, whatever mechanism leads to loss-of-function of the studied ENaC mutations, the synthetic peptides solnatide and AP318 could restore ENaC function of conserved PHA1B mutants to at least current levels of WT ENaC. This implies that the TIP domain activates ENaC by some mechanism which remains intact even in the presence of various mutations occurring in different subunits, because binding to the putative binding site in the carboxyl terminal domain of the glycosylated α-subunit apparently remains basically unaffected in all tested point mutations or was compensated in frameshift mutations via a moderate activation of αβ- and βγ-ENaC, respectively. Collier et al. (2014) showed that residues located in the wrist region (which lies above the transmembrane domain) at equivalent positions at the interface of the thumb and palm domains in α-, β-, and γ-subunits, form intersubunit interfaces between which conformational changes are critical to ENaC gating. Cross-linking of these residues alters ENaC activity. Long cross-linkers increase ENaC current, whereas short cross-linkers reduce ENaC open probability. The current activating effect of TIP peptides in all PHA1B mutants could be explained by their interfering with conformational changes between subunit interfaces in the wrist region such as to increase intersubunit distance and thereby channel open probability. In any case, as therapy of PHA1B is only symptomatic so far, the TIP peptides solnatide and AP318, which directly target ENaC, are promising candidates for the treatment of the channelopathy caused disease PHA1B.

## Author contributions

AW gave substantial contribution to the design of the work, performed experiments, analyzed and interpreted data, drafted the work and approved the version to be published. MA performed experiments, analyzed and interpreted data, drafted the work and approved the version to be published. ST gave substantial contributions to the conception and design of the work, interpretation of data, drafted the work and approved the version to be published. HE, SC, SG performed experiments, analyzed data, drafted the work and approved the version to be published. BF, HF, HP gave contribution to the conception of the work, revised it critically and approved the version to be published. IC, RL interpreted data, revised the work critically for important intellectual content and approved the version to be published. RLG gave substantial contribution to the conception and design of the work, interpretation of data, drafted the work and approved the version to be published. WS gave substantial contribution to the design of the work, performed experiments, analyzed data, drafted the work and approved the version to be published. All authors agree to be accountable for the content of the work.

## Funding

This work was funded by Wellcome Trust Pathfinder Award 105632. AW and WS also received financial support from APEPTICO R&D Vienna, Austria. RL was supported by an NIH RO1 DK100564 RO1 grant and IC received an AHA postdoctoral award 15POST22820021.

### Conflict of interest statement

The authors declare that the research was conducted in the absence of any commercial or financial relationships that could be construed as a potential conflict of interest.

## Abbreviations

ASIC1: acid-sensing ion channel subunit 1
CRDs: cysteine-rich domains
ENaC: epithelial sodium channel
ENaC/DEG: ENaC/degenerin family
HEK-293: human embryonic kidney
PHA1B: pseudohypoaldosteronism type 1B
PNGase F: peptide-*N*^4^-(*N*-acetyl-β-D-glucosaminyl)asparagine amidase F
peptide: *N*-glycanase
SGK-1: serum- and glucocorticoid-regulated kinase 1
TM: transmembrane
TNF: tumor necrosis factor
WT: wild type.
